# Transcriptome Analysis and Identification of Genes Associated with Floral Transition and Flower Development in Sugar Apple (*Annona squamosa* L.)

**DOI:** 10.3389/fpls.2016.01695

**Published:** 2016-11-09

**Authors:** Kaidong Liu, Shaoxian Feng, Yaoling Pan, Jundi Zhong, Yan Chen, Changchun Yuan, Haili Li

**Affiliations:** Life Science and Technology School, Lingnan Normal UniversityZhanjiang, China

**Keywords:** *A. squamosa*, flower development, transcriptome, digital gene expression, phytohormone, circadian rhythm

## Abstract

Sugar apple (*Annona squamosa* L.) is a semi-deciduous subtropical tree that progressively sheds its leaves in the spring. However, little information is available on the mechanism involved in flower developmental pattern. To gain a global perspective on the floral transition and flower development of sugar apple, cDNA libraries were prepared independently from inflorescent meristem and three flowering stages. Illumina sequencing generated 107,197,488 high quality reads that were assembled into 71,948 unigenes, with an average sequence length of 825.40 bp. Among the unigenes, various transcription factor families involved in floral transition and flower development were elucidated. Furthermore, a Kyoto Encyclopedia of Genes and Genomes pathway enrichment analysis revealed that unigenes exhibiting differential expressions were involved in various phytohormone signal transduction events and circadian rhythms. In addition, 147 unigenes exhibiting sequence similarities to known flowering-related genes from other plants were differentially expressed during flower development. The expression patterns of 20 selected genes were validated using quantitative-PCR. The expression data presented in our study is the most comprehensive dataset available for sugar apple so far and will serve as a resource for investigating the genetics of the flowering process in sugar apple and other *Annona* species.

## Introduction

Sugar apple (*Annona squamosa* L.) is an important member of the *Annonaceae* family, which consists of ~166 genera and more than 2000 species (Höllerhage et al., 2015). As a commercially important fruit tree, sugar apple orchards are wide spread and well adapted to the edaphoclimatic conditions of tropical and subtropical zones. Due to its medicinal and nutritional properties, sugar apple tree is well known, and widely planted throughout the tropics (Gupta et al., 2015; Liu et al., 2015). The axillary buds of sugar apple are cryptic, obscured by the base of the petiole, and released following leaf abscission. Summer defoliation promotes flowering by releasing dormant subpetiolar axillary buds and causes the resumption of flower development (Olesen and Muldoon, 2009). The flowers are born terminally on the new shoots. However, improper shoot state, low temperature, or short day conditions may cause the unreliable flowering of sugar apple (Olesen and Muldoon, 2012; Liu et al., 2015).

Flower development is the most important developmental event in the life cycle of higher plants, especially fruit trees. As an intricate biological and morphological process, flower development is regulated by a large number of genes (Huang et al., 2013). Flowering is the first step of sexual reproduction, and the timing of the transition from vegetative growth to flowering is an important characteristic in agriculture and horticulture (Bernier et al., 1993; Zhang et al., 2013). Many specifically expressed transcription factor (TF) genes, including *SUPPRESSOR OF OVEREXPRESSION OF CO 1* (*SOC1*), *FLOWERING LOCUS T* (*FT*), *CONSTANS* (*CO*), *AGAMOUS-LIKE 24, FLOWERING LOCUS D, FLOWERING LOCUS E, FLOWERING WAGENINGEN*, and *PROTODERMAL FACTOR2*, were identified during the flowering process, suggesting a complex gene regulatory network underlying flower development (Matías-Hernández et al., 2016). Subsequently, these floral integrators trigger floral meristem identity genes *LEAFY* and *APETALA1* to promote flowering (Komeda, 2004).

Genetic and physiological studies have demonstrated that the timing of the floral transition is controlled by six major pathways, photoperiod, gibberellin (GA)-mediated, vernalization, autonomous, thermosensory, and aging, which trigger or repress the changes, from the generation of leaves to the development of reproductive organs, in the shoot meristem (Blázquez et al., 2003; Srikanth and Schmid, 2011; Yamaguchi and Abe, 2012). In model plants, photoperiod, the change of daylength over the year, is a major determinant of floral transition (Andrés and Coupland, 2012). Photoperiod is detected by the leaves and thus a signal has to transmit from the shoot apex to initiate flowering (Johansson and Staiger, 2015). The requirement for exposure to long-term cold to initiate the floral transition is known as vernalization. Addition to photoperiod, vernalization is also required to prevent flowering in winter, but permits flowering in spring (Kim et al., 2009). Besides, the flowering response to temperature relies on the thermosensory pathway, which is important for mitigating the effects of temperature change (Kumar et al., 2012). Recent studies showed that GAs function not only to promote the growth of organs, but also to induce phase transitions of flowering. The involvement of GAs in floral initiation and development has been uncovered in many plant species (Mutasa-Göttgens and Hedden, 2009). In addition to GA, other endogenous hormones, as both positive and negative elements, also serve within the signal network leading to a reproductive phase transition, depending on the phytohormones and growth conditions (Davis, 2009; Domagalska et al., 2010). In apple, complex hormone regulatory networks involved in cytokinin (CK), abscisic acid (ABA) and GA pathways also induce flower formation (Xing et al., 2015). In *Agapanthus praecox* ssp. *orientalis*, GA signaling regulates the scape elongation and stimulates early-flowering, while indoleacetic acid (IAA) signaling delays flowering slightly (Zhang et al., 2014a).

The flowering mechanism has been studied well in the model plant *Arabidopsis thaliana* and to some extent in a few other plant species (Melzer et al., 2008; Andrés and Coupland, 2012; Song et al., 2012). However, the information available about the molecular basis of floral initiation and differentiation in the genus *Annona* is very scarce. To permit the genetic engineering of this important agronomic trait, it is imperative to gain an understanding of the molecular basis of flower development in sugar apple.

Without a reference genome, *de novo* sequencing using Illumina short RNA-sequencing (RNA-seq) reads is the most cost effective approach to generate a large collection of expressed sequence tags (ESTs) for subsequent analyses. Transcriptome assembly has been successfully applied to many fruit trees, such as pear, melon, litchi, and Chinese cherry (Bai et al., 2013; Corbacho et al., 2013; Lu et al., 2014; Zhu et al., 2015). In our study, we have constructed independent cDNA libraries of four different flower stages in *A. squamosa* for Illumina RNA-seq. The annotation of transcriptome sequences and analysis of the expression profiles of differential genes have provided valuable genomic sources for future research regarding the flowering mechanism of *A. squamosa*. Furthermore, the kinetics of the expression of the expression patterns of hormone-related genes and the changes of hormone contents in *A. squamosa* were also have been revealed. Our data will aid understanding of the involvement of hormonal signaling in flowering mechanism for woody plants in general.

## Materials and methods

### Plant material and experiment procedures

Commercially cultivated adult trees (10–12-years-old) of *A. squamosa* cv. “Bendi” were selected. The trees were planted in a 4 × 4 m arrangement with drip irrigation and fertilizer applications as required. Trees were located at the Ling Nan Normal University field experimental station in Zhanjiang City (Guangdong Province, China) at 21°7′ 36″ N latitude, 110° 14′ 24″ E longitude, and an altitude of 21.34 m above sea level. The inflorescent meristem (IM), the flower buds (FB), and two stages of flowers (FL1 and FL2) were collected from *A. squamosa* trees. The flower buds were collected based on their size (3–6 mm). The two flower stages were the mature flowers with partially opened petals (FL1) and mature flowers with opened and faded petals (FL2). These tissue samples were frozen immediately in liquid nitrogen, and stored at −80°C until use.

### RNA isolation and library preparation

Total RNA was extracted using the TRIzol Kit (Promega, Beijing, China) according to the manufacturer's protocol. Equal volumes of RNA from each of the four stages of flower development were pooled. Each sample was prepared by mixing three replicate samples. Then, the total RNA was treated with RNase-free DNase I (Takara, Dalian, China) for 30 min at 37°C to remove residual DNA. RNA quality was verified by RNase-free agarose gel electrophoresis and the total RNA concentration was measured using a 2100 Bioanalyzer (Agilent Technologies, Santa Clara, USA) at 260 nm and 280 nm. RNA samples with 260/280 nm ratios between 1.8 and 2.0 were used for subsequent analyses.

The transcriptome assembly library, as a reference library, was constructed by mixing equal amounts of RNA from the above four samples. Briefly, total mRNA was isolated with oligo (dT) cellulose. All of the mRNA was broken into short fragments (200 nt) by adding the fragmentation buffer. First-strand cDNA was generated using random hexamer-primed reverse transcription. Second-strand cDNA was synthesized by DNA polymerase I and RNase H. Then, the synthesized cDNA fragments were purified and then, subjected to end pairing, the addition of a single “A” bases, and ligation with Illumina adapters. The ligation products were size fractioned by agarose gel electrophoresis, and fragments were excised for PCR amplification. The amplified fragments were sequenced using Illumina HiSeq™ 2500 by Gene Denovo Co. (Guangzhou, China).

### *De novo* assembly and functional annotation

For *de novo* assembly, reads with more than 5% N bases (bases unknown) and those containing adaptor sequences were removed. Low quality reads containing more than 20% of low *Q* (≤ 10) bases were also removed. Then, the clean reads were assembled using Trinity to construct unique consensus sequences (Grabherr et al., 2011). The raw sequence data has been submitted to the NCBI Short Read Archive with accession number SRA423630. The assembled unigenes were aligned to a series of protein databases using the BLASTX alignment algorithm with *E* < 0.00001. These databases include the NCBI Nr protein database (http://www.ncbi.nlm.nih.gov), Swiss-Prot protein database (http://www.expasy.ch/sprot), KEGG pathway database (http://www.genome.jp/kegg) and COG database (http://www.ncbi.nlm.nih.gov/COG). The sequence direction of the unigenes was assigned according to the best alignment from the four databases. When the results conflicted among databases, then the following order of priority was employed: NR, Swiss-Prot, KEGG, and COG. Blast2GO was used to produce the Gene Ontology (GO) (http://www.geneontology.org/) annotation results for unigenes. The functional classification of unigenes was performed using WEGO software (http://wego.genomics.org.cn/cgibin/wego/index.pl). KEGG pathway annotation was performed by BLASTall software against the KEGG database.

### Analysis and mapping of digital gene expression (DGE) tags

To map DGE tags, sequencing-related raw image data were filtered to remove low quality tags (tags with unknown “N” sequences), empty tags (sequence with only the adaptors but no tags), and tags with only one copy number (which might result from sequencing errors). For annotation, cleaned tags containing CATG, and the 21-bp tag sequence were mapped to our transcriptome reference database with no more than 1 nucleotide mismatch. All of the tags that mapped to the reference sequences of multiple genes were filtered out, and the remaining tags were designated as unambiguous tags for gene expression analysis. The number of unambiguous tags of each gene was calculated and then normalized to the number of transcripts per million clean tags. The differentially expressed tags were used for mapping and annotation.

### Screening of differentially expressed genes (DEGs)

The alignment software, Bowtie 0.12.8, was used to map the reads to the transcriptome. To compare the differences in gene expression at different flower developmental stages, the tag frequencies in the different DGE libraries were statistically analyzed. The number of mapped clean reads for each unigene was then counted and normalized into a Reads Per kb per Million reads (RPKM) to calculate unigene expression. A false discovery rate <0.001 and an absolute value of log2 ratio >1 were used as thresholds to determine the significant differences in gene expression levels. The DEGs were used for GO and KEGG enrichment analyses using a method similar to that described in previous studies (Zhang et al., 2013). GO terms, which take the corrected *P* ≤ 0.05 as a threshold, are significantly enriched in DEGs. KEGG pathways with *Q* ≤ 0.05 are significantly enriched in DEGs

### Real-time PCR validation

First-strand cDNA was generated from 1 μg total RNA isolated from each of the four flower developmental stages using the Superscript first-strand synthesis system (Invitrogen, Shanghai, China). Primers for quantitative reverse transcription PCR (qRT-PCR) were designed using Primer Premier 5.0 software (Premier Biosoft International, Palo Alto, CA, USA) and synthesized by Sangon Biotech (Shanghai) Co., Ltd. qRT-PCR was performed on a Bio-Rad iQ5 Optical System Real Time PCR System (Bio-Rad, Hercules, CA, USA) using a SYBR Green-based PCR assay. Each reaction mixture was 20 μL, containing 6 μL of diluted first-strand cDNAs, 250 nM of each primer, and 10 μL of SYBR Green PCR Master Mix (TaKaRa, Japan). The qPCRs were run as follows: 50°C for 2 min, 95°C for 10 min, followed by 40 cycles of 95°C for 30 s, 56°C for 30 s, and 72°C for 30 s in 96-well optical reaction plates. The sugar apple *Actin* gene was used as an internal standard to calculate relative fold-differences based on comparative cycle threshold (2^−ΔΔ*Ct*^) values.

### Standard curves and efficiency of amplification

The limit of detection and the amplification efficiency of the qRT-PCR were determined using 10-fold serial dilution of cDNA isolated from one sample (leaves), which was used to create the standard curve. The slopes and correlation coefficients of the standard curves were used to calculate the PCR efficiency (E) of primer pairs. In our experiment, the *E*-value each primer pair was calculated by formula: E = POWER (10, 1/slope)^−1^. The value of E for each primer pair was between 0.9 and 1.1. In our study, 1 μl of cDNA (30 ng/μL) from different experiment samples were used as temples for qRT-PCR analysis.

### Measurements of various hormones

For the exogenous hormone contents analysis, individual samples from different developmental stages of sugar apple flowers were harvested and then immediately frozen in liquid nitrogen and stored at −80°C until extraction. Each sample was prepared by three replicate samples. The inflorescent meristem (IM), the flower buds (FB) and two stages of flowers (FL1 and FL2) were consistent to the ones for RNA sequencing in order to link the DEG to hormone contents. Endogenous GA was detected by nano-LC-ESI-Q-TOF-MS analysis as described previously (Chen et al., 2012). The exogenous IAA contents were determined using a FOCUS GC-DSQII (Thermo Fisher Scientific Inc., Austin, TX, USA; Shen et al., 2014). Additionally, ABA and Cytokinin (ZRs) were detected using a UFLC-MS/MS system as described Kasote et al. (2016).

### Statistical analysis

Significant differences between values were calculated using a one-way ANOVA analysis with a Tukey test at a significance level of α = 0.01 in Excel software. All expression analyses were performed for five biological replicates. All reported values represent arithmetic averages of five replicates, and data are expressed as mean plus or minus standard deviation (mean ± SD).

## Results

### Sequencing, assembly and annotation of a sugar apple reference transcriptome

To obtain a reference transcriptome for sugar apple flowers, RNA-seq libraries were constructed using RNA samples from four different flower stages. In total, 107,197,488 raw reads were obtained from four different flower samples. After removing the low-quality reads, all of the clean reads from the four RNA-seq data sets were combined and used for transcript assembly (Table S1). The Trinity package assembled 71,948 unigenes, with an average size of 825.40 nt (Table S2). The size distributions of these unigenes are shown in Figure S1.

**Figure 1:**
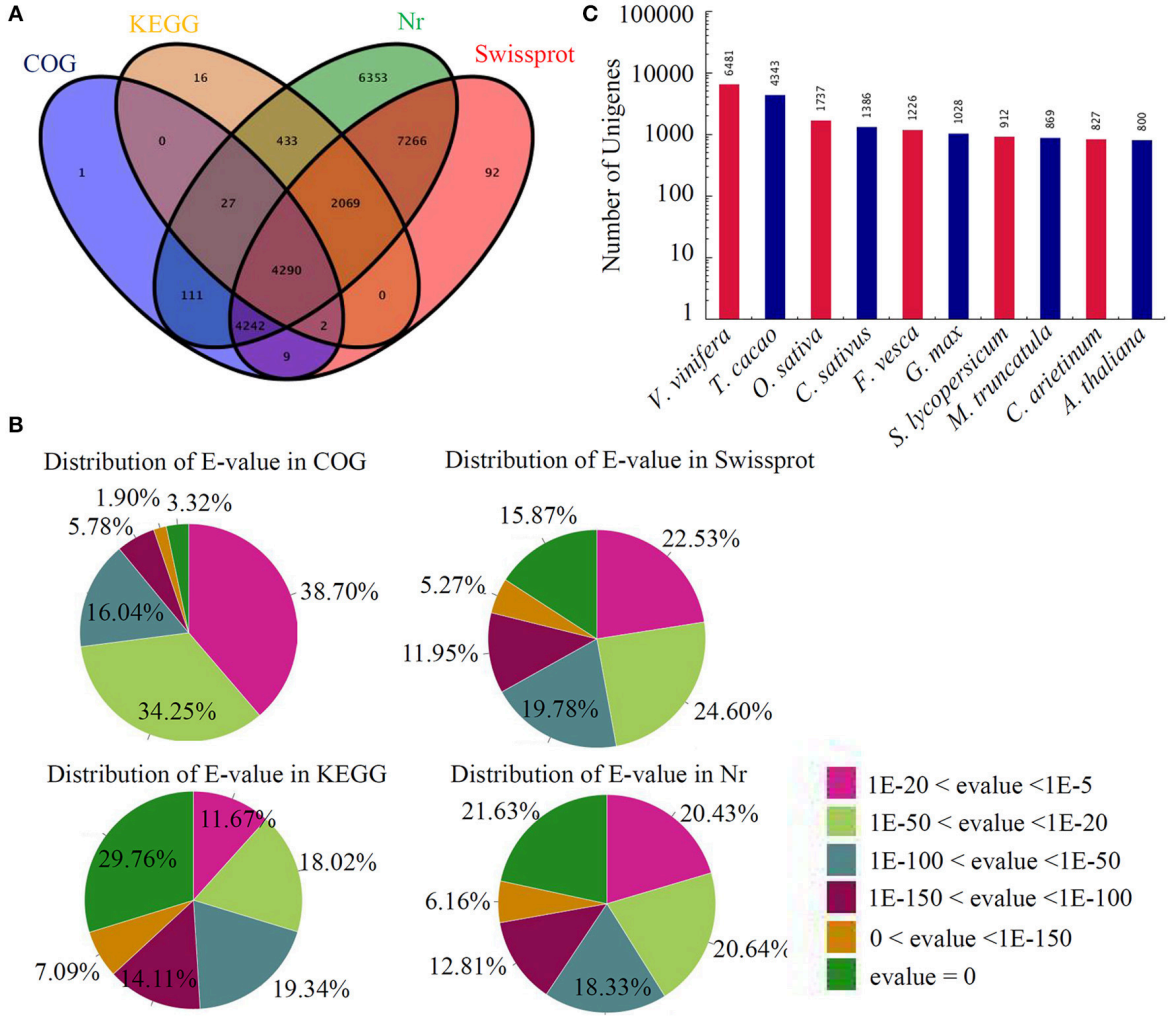
**Annotation of assembled sugar apple unigenes. (A)** In total, 24911 unigenes were annotated by different protein databases. The number of unigenes annotated by different databases, including Nr, Swissprot, COG, and KEG, were showed in a Venn diagram. **(B)** Distribution of *E*-value in Nr database. **(C)** Identification of the transcripts of other plant species homologous to the annotated unigenes of sugar apple.

Next, the BLAST algorithm was utilized to identify the transcripts of other organisms homologous to the assembled unique genes of sugar apple. In summary, 24,911 unigenes were annotated by BLASTX (*E* < 1e^−5^) using the NCBI nr database, while 17,970 were annotated using the Swiss-Prot protein database. In addition, 6837 and 8682 unigenes could be annotated according to the Kyoto Encyclopedia of Genes and Genomes (KEGG) and Cluster of Orthologous Groups of protein (COG) databases, respectively. Approximately 6% (4290) of unigenes could be assigned to a homolog in all four databases (Figure 1A). The distributions of *E*-values in different databases are showed in Figure 1B. A large number of unigenes in sugar apple showed high similarities to the genes in other plant species. The numbers of homologous genes in the top 10-hit species are shown in Figure 1C. Interestingly, the largest number of sugar apple homologous genes was identified in *Vitis vinifera*, suggesting a close relationship between these two fruit trees.

### Classification of GO and KEGG terms

We further assigned gene ontology (GO) terms to sugar apple unigenes. A total of 12,351 unigenes could be classified into the three GO categories: biological process, cellular component, and molecular function. Within the biological process category, the most highly represented terms were “cellular process,” “metabolic process,” and “response to stimulus.” Within the molecular function category, “catalytic activity,” and “binding” were the two most abundant terms. The largest terms within the cellular component category were “cell,” “cell part,” and “organelle” (Figure S2)

To further determine the involvement of metabolic pathways in the flowering process, we predicted the KEGG pathways represented by all of the assembled unigenes. A total of 6837 unigenes from four samples were mapped into 124 KEGG pathways. The maps with the highest unigene representation were metabolic pathway (ko01100) with 1892 unigenes, followed by biosynthesis of secondary metabolites (ko01110), ribosome (ko03010), protein processing in endoplasmic reticulum (ko04141), starch and sucrose metabolism (ko00500), oxidative phosphorylation (ko00500) and plant-pathogen interaction pathway (ko04626) (Table S3).

### Transcriptome dynamics during flower development

**Figure 2:**
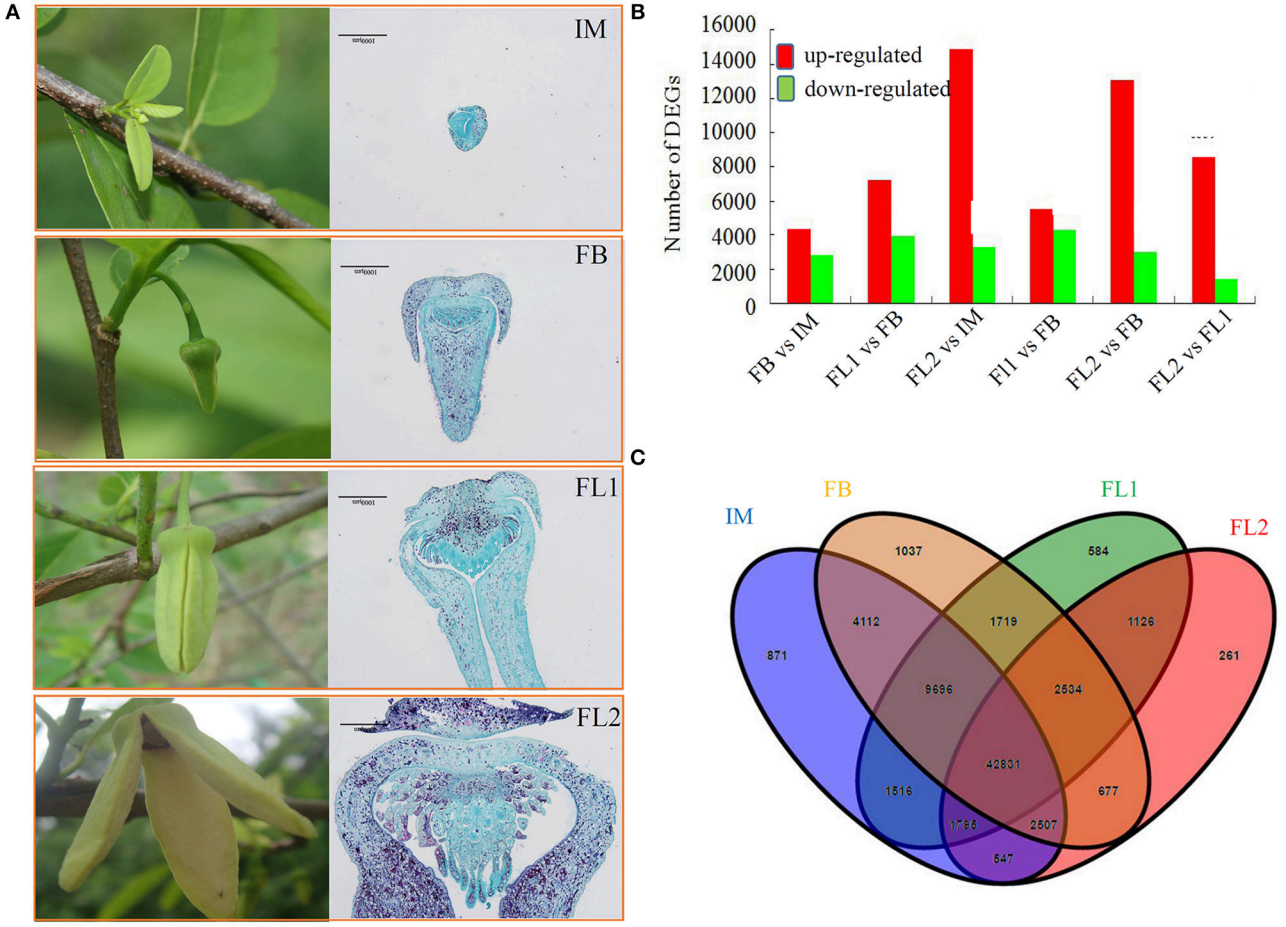
**Analysis of the differentially expressed unigenes (DEGs) during the floral transition and flower development process in sugar apple. (A)** Numbers of DEGs in different comparisons, including FB vs. IM, FL1 vs. FB, FL2 vs. IM, FL1 vs. FB, FL2 vs. FB, and FL2 vs. FL1. The red indicated up-regulated unigenes and green indicated down-regulated unigenes. **(B)** Venn diagram showed the number of DEGs in different stages of flower development. **(C)** The expression profiling of stage-preferential unigenes in sugar apple.

To study the transcriptome dynamics and identify the2 candidate genes involved in flower development, four transcriptomes were generated from different flower samples (IM, FB, FL1, and FL2). We compared the transcript levels of each unigene between different samples. In the FB vs. IM comparison, 7227 differentially expressed, 4366 up-regulated and 2861 down-regulated, transcripts were detected. In the FL1 vs. FB comparison, 11,070 differentially expressed, 7169 up-regulated and 3901 down-regulated, transcripts were found. In the FL2 vs. IM comparison, 14,883 DEGs were up-regulated and 3261 DEGs were down-regulated. In the FL1 vs. IM comparison, 9729 differentially expressed, 5481 up-regulated and 4248 down-regulated, transcripts were indentified. In the FL2 vs. FB comparison, 18,144 differentially expressed, 13,109 up-regulated and 2985 down-regulated, transcripts were uncovered. Lastly, in the FL2 vs. FL1 comparison, 8539 DEGs were up-regulated and 1386 DEGs were down-regulated. (Figure 2A)

The overlaps among samples from independent flower stages were calculated and shown using a Venn diagram. Approximately 83.43% of the assembled unigenes were shared by at least three libraries made from the different flower developmental stages. In detail, 871 and 1037 DGEs were only in the IM and FB libraries, respectively; while 584 transcripts were mainly expressed in the FL1 stage. Moreover, 261 transcripts were mainly expressed in the FL2 stage. The overlap between IM and FB consisted of 4112 (5.73%) single unigenes. Similarly, 1126 transcripts were found in the overlapping regions of FL1 and FL2 (Figure 2B).

To identify flower developmental stage-preferential expressed genes, DGE libraries were analyzed using criteria of 2-fold differences and adjusted *P* < 0.05. The largest number of stage-preferential genes was identified in the IM stage (2957 unigenes) (Figure 2C, Figure S3).

### Cluster analysis of DEGs during the flower development process in sugar apple

**Figure 3:**
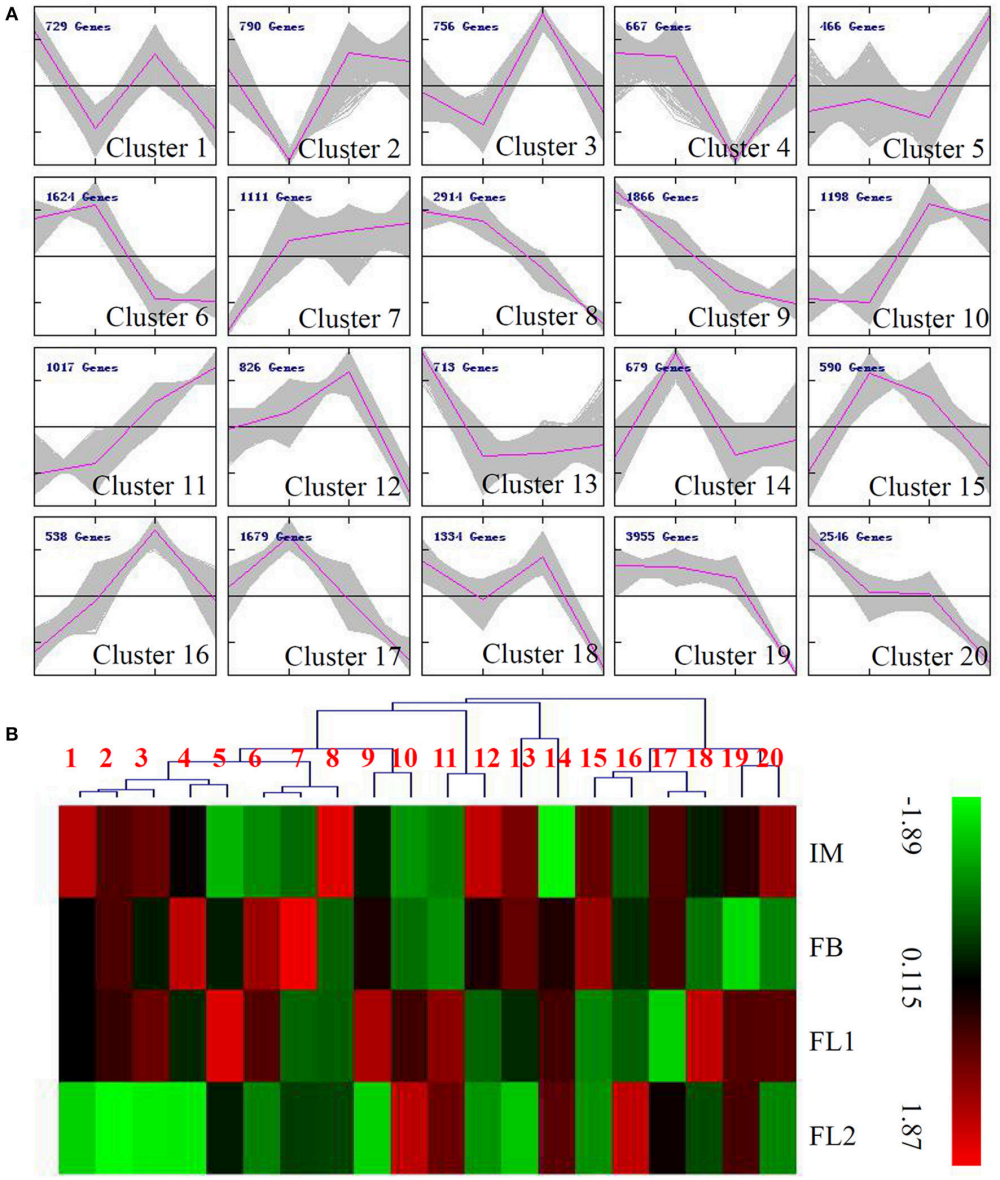
**Expression profiles of the differentially expressed unigenes during the flowering process in sugar apple. (A)** MeV cluster analysis of differentially expressed unigenes from the expression profiles during the flowering process. Red lines indicated the average expression level of unigenes grouped into the same Cluster under different flowering stages. **(B)** Heat map for cluster analysis of the differentially expressed unigenes by K-means method. Red indicates up-regulated genes and blue indicates down-regulated genes.

To reflect the major trends and the key transitional states (IM, FB, FL1, and FL2) during the flower development process in sugar apple, all 25,998 DEGs were assigned to 20 clusters by the K-means method. The expression levels of the genes belonging to clusters 1, 7, and 11 increased during the flowering process; while the expression levels of genes belonging to clusters 8, 9, 19, and 20 decreased during the flowering process. The unigenes showing IM stage-specific expression levels were grouped into clusters 9, 13, and 20, while the unigenes showing FB stage-specific expression levels were grouped into clusters 14, 15, and 17. The unigenes classed into clusters 3, 12, and 16 were highly expressed in the FL1 stage, and the transcript levels of unigenes in clusters 5 and 11 were very high in the FL2 stage (Figure 3A). The average expression levels of the unigenes belonging to each cluster are shown in a heat map in Figure 3B.

### Identification of the flower development-associated TF-encoding genes

**Figure 4:**
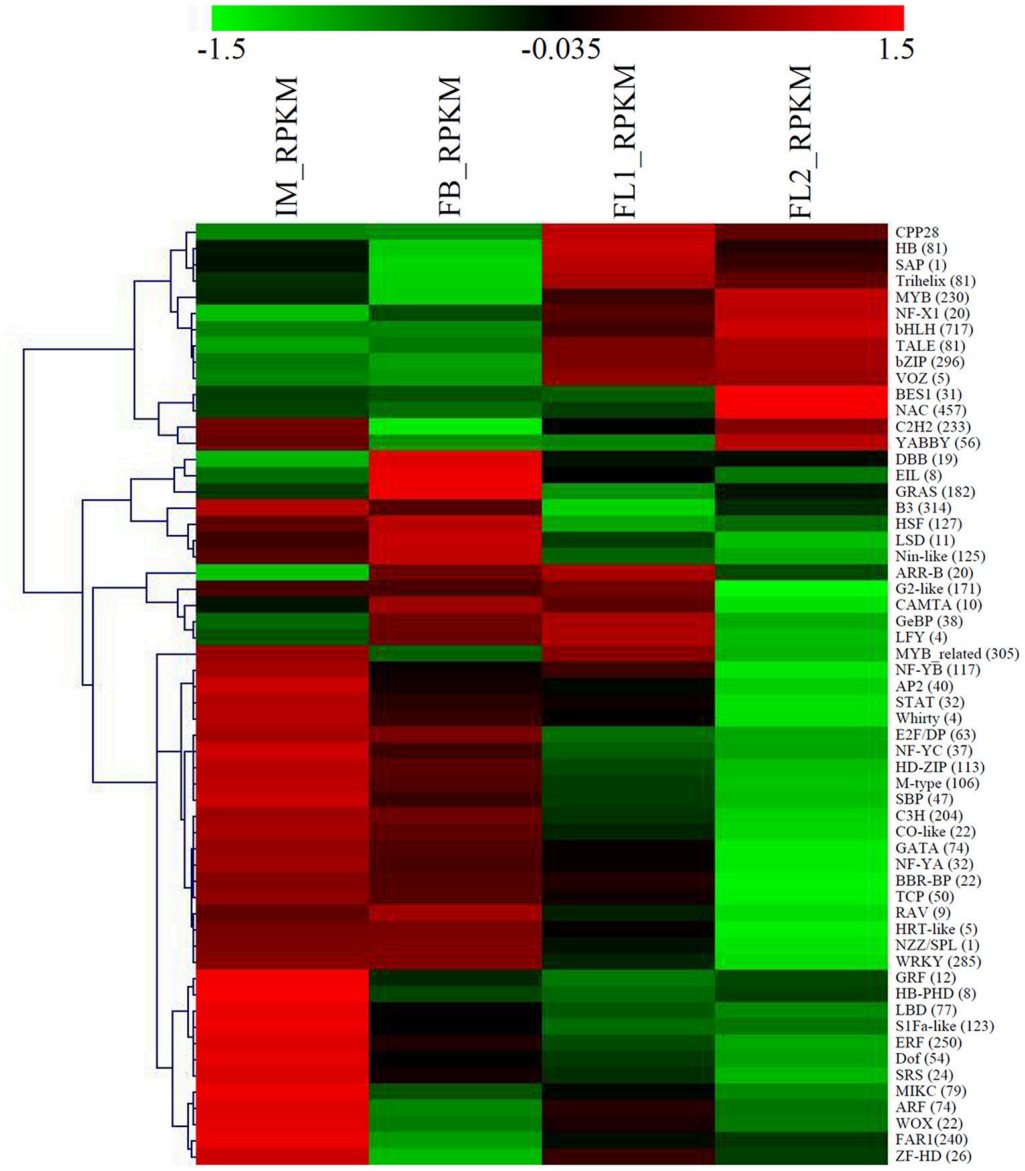
**Identification and analysis of floral transition and flower development-associated transcription factor genes**. A heat map depicting the overall trend of the differential expression profiles of the transcription factor genes during flower development was constructed using MeV. The number of each transcription factor was showed in parentheses.

TFs are key regulatory proteins of transcription in biological processes, especially in flower development (Stewart et al., 2016). Therefore, we studied the expression dynamics of TF genes in sugar apple. In total, 5903 TF genes were identified in the flower bud and flower development stages. The basic helix-loop-helix (bHLH; 717 members), NAC (457 members), B3 (314 members), MYB-related (305 members), basic leucine zipper (bZIP; 296 members), WRKY (285 members), ERF (250 members), FAR1 (240 members), C2H2 (233 members), and MYB (230 members) families were identified as the top 10 largest families during flower development, and some are critical components of plant adaptive responses to biotic and abiotic stresses, and senescence. A heat map depicting the overall expression trend of the TF genes during flower development was constructed using MeV software. Some TF families were significantly up-regulated. For example, the bHLH, MYB, and bZIP families were significantly up-regulated after the FL1 stage; while the NAC and C2H2 families were up-regulated after the FL2 stage. By contrast, more TF families, such as WRKY, ERF, and FAR, were significantly down-regulated (Figure 4).

### Identification of flowering time-associated and flower development-related genes

Based on a comparative analysis of the NCBI and UniProt databases, 144 unigenes in sugar apple showed homology to known flowering time-associated genes from other plant species (Table S4). Most of the flowering time-associated genes in sugar apple could be assigned to six classical flowering-related pathways.

**Figure 5:**
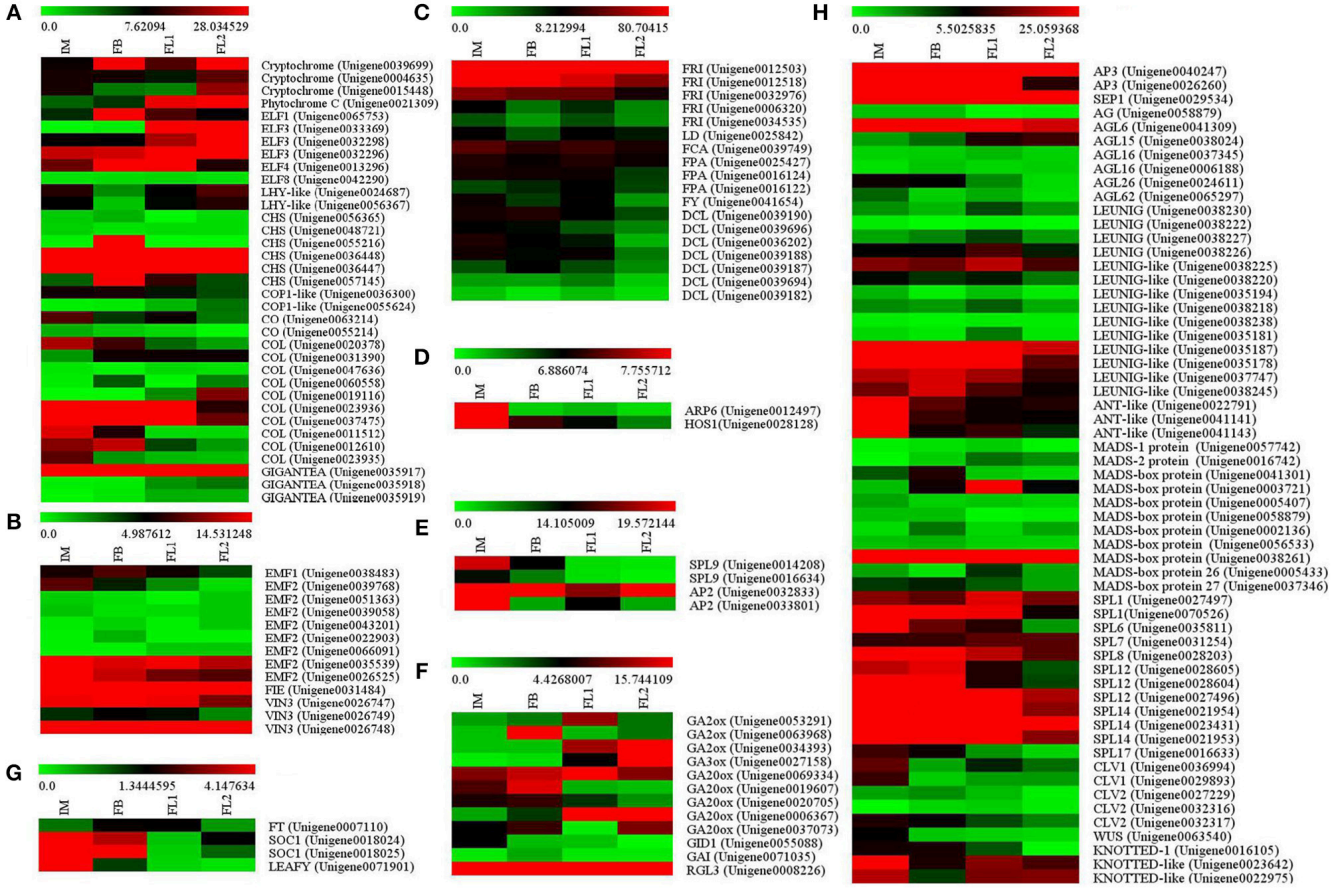
**Identification of the floral transition and flower development-associated genes. (A)** Flower development stage-preferential expression pattern of the genes involved in photoperiod pathway. **(B)** Flower development stage-preferential expression pattern of the genes involved in autonomous pathway. **(C)** Flower development stage-preferential expression pattern of the genes involved in vernalization pathway. **(D)** Flower development stage-preferential expression pattern of the genes involved in thermosensory pathway. **(E)** Flower development stage-preferential expression pattern of the genes involved in aging pathway. **(F)** Flower development stage-preferential expression pattern of the genes associated with flowering integron. **(G)** Flower development stage-preferential expression pattern of the genes involved in GA signaling pathway. **(H)** Flower development stage-preferential expression pattern of the genes related to non-classified flowering regulators.

In the photoperiod pathway, several sugar apple unigenes showed homology to the components of *PHYTOCHROME* (*PHY*, 1 unigenes), *CRYPTOCHROME* (*CRY*, 3 unigenes), *GIGANTEA* (*GI*, 3 unigenes), *EARLY FLOWERING* 1 (1 unigene), *EARLY FLOWERING* 3 (*ELF3*, 3 unigenes), *EARLY FLOWERING* 4 (*ELF4*, 1 unigene), *EARLY FLOWERING* 8 (*ELF8*, 1 unigene), and *CHALCONE SYNTHASE* (*CHS*, 6 unigenes) (Figure 5A, Table S4). In addition, several sugar apple unigenes exhibited similarities to genes in the vernalization pathway, including *EMBRYONIC FLOWER* 1 (*EMF1*, 1 unigene), *EMBRYONIC FLOWER* 2 (*EMF2*, 8 unigenes), *FERTILIZATION INDEPENDENT ENDOSPERM* (*FIE*, 1 unigene), and *VERNALIZATION INSENSITIVE* 3 (*VIN3*, 3 unigenes) (Figure 5B, Table S4). Moreover, 18 unigenes in sugar apple showed high similarities to genes that are involved in the autonomous pathway, such as *FRIGIDA* (*FRI*, 5 unigenes), *LUMINIDEPENDENS-like* (*LD*), *FLOWERING TIME CONTROL PROTEIN FCA* (*FCA*), *FLOWERING TIME CONTROL PROTEIN FPA* (*FPA*), *FLOWERING TIME CONTROL PROTEIN FY* (*FY*), and *DICER-LIKE* (*DCL*) (Figure 5C, Table S4). Putative thermosensory pathway unigenes were identified, including an *ACTIN RELATED PROTEIN 6* (*ARP6*) and a *HIGH EXPRESSION OF OSMOTICALLY RESPONSIVE GENES 1* (*HOS1*) (Figure 5D; Table S4). For the aging pathway, many unigenes were found, including *APETELA2* (*AP2*) and *SQUAMOSA PROMOTER-BINDING-LIKE PROTEIN 9* (*SPL9*) (Figure 5F; Table S4). Some putative homologous genes of the GA pathway were identified, including *GA2 oxidase* (*GA2ox*, 3 unigenes), *GA3 oxidase* (*GA3ox*, 1 unigene), *GA20 oxidase* (GA20ox, 5 unigenes), the GA receptor DELLA protein *GIBBERELLIC ACID INSENSITIVE1* (*GAI*, 1 unigene), and the GA receptor *GIBBERELLIN INSENSITIVE DWARF1* (*GID1*, 1 unigene) (Figure 5F; Table S4).

In addition to the unigenes related to the six pathways, we obtained some unigenes associated with flowering integron (FT and SOC1), and one unigene related to floral meristem identity (*LEAFY*) (Figure 5G; Table S4). Some additional non-classified flowering regulators, including members of the MADS transcription factor, ANT-like transcription factor and SQUAMOSA PROMOTER-BINDING PROTEIN, were also identified (Figure 5H; Table S4).

### Identification of unigenes involved in the circadian rhythm pathway

**Figure 6:**
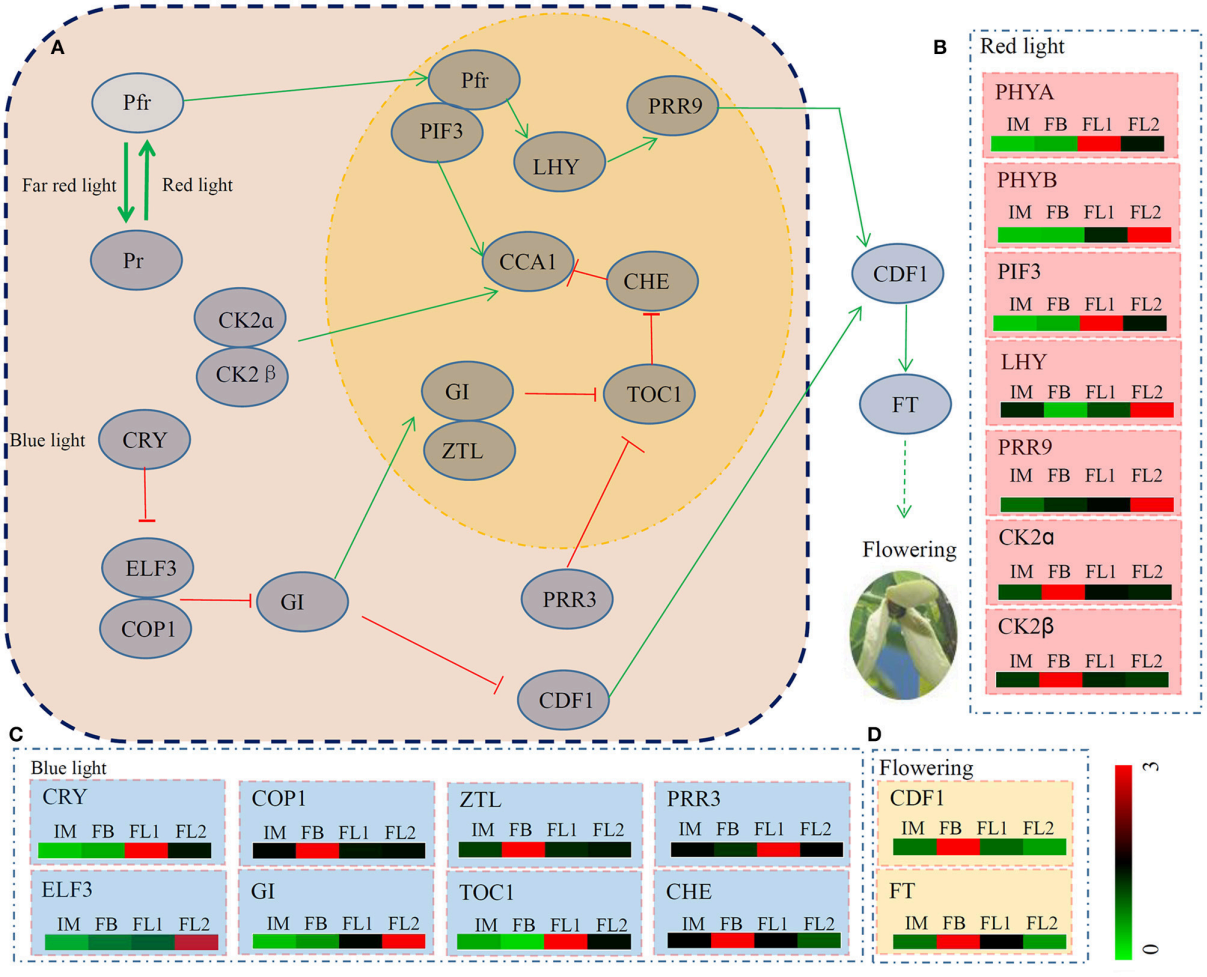
**The detailed information on genes involved in the pathway of circadian rhythm. (A)** The putative regulation network of circadian clock associated with flowering in sugar apple. Red arrow indicated depressing; and green arrow indicated activating. **(B)** The expression pattern of red light signaling pathway related genes during the flowering process. **(C)** The expression pattern of blue light signaling pathway related genes during the flowering process. **(D)** The expression pattern of two key flower development regulation genes during the flowering process. The different colors correspond to the log-transcription values of RPKM refer to each gene shown in the bar at the lower right corner of the figure.

Pathway analyses of our transcriptome data showed that many unigenes were involved in multiple metabolic pathways. Determined by photoreceptors, circadian rhythms are a key regulatory system in controlling plant flowering (Izawa et al., 2002; Jiao et al., 2005). In total, 17 key regulators encoded by 39 unigenes identified four of our transcriptomes (Figure 6A; Table S5). The average expression levels of the unigenes associated with each key regulator were also counted based on the RPKM values. The genes encoding seven regulators, CK2α, CK2β, COP1, ZTL, CDF1, CHE and FT, were predominantly expressed in the FB stage. The genes encoding five regulators, PHYA, PIF3, CRY, PRR3 and TOC1, were highly expressed in the FL1 stage. The genes encoding another five regulators, PHYB, LHY, PRR9, EARLY FLOWERING 3, and GI, showed their highest expression levels in the FL2 stage. Interestingly, no genes encoding any of the regulators were highly expressed in the IM stage (Figures 6B–D).

### Identification of unigenes involved in the pathways of various flowering-related hormones

Several phytohormones, including auxin, ABA, GA and cytokinin, are closely correlated to flower transition and development (Heisler et al., 2005; Davis, 2009; Bartrina et al., 2011). To investigate the relationship between phytohormones and flower development in sugar apple, the expression changes of unigenes involved in phytohormone signaling pathways were analyzed.

**Figure 7:**
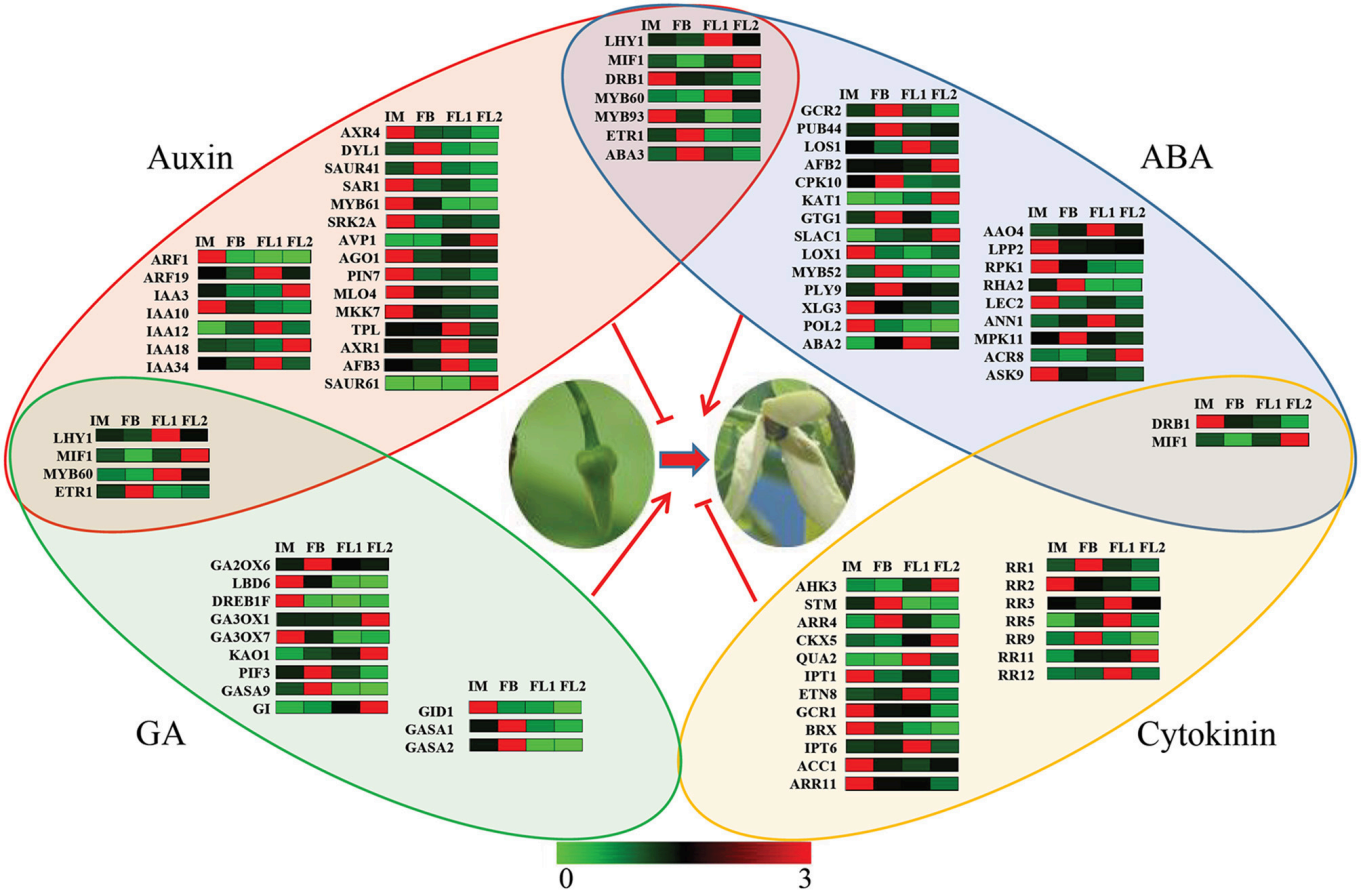
**Network analysis of various hormones involved in the regulation of flowering process**. The red shadow color indicated the key genes associated with auxin signaling pathway. The blue shadow color indicated the key genes with ABA signaling pathway. The green shadow color indicated the key genes with GA signaling pathway. The yellow shadow color indicated the key genes with cytokinin signaling pathway. The different colors correspond to the log-transcription values of RPKM refer to each gene shown in the bar at the bottom of the figure.

In total, 32 key regulators in the ABA-signaling pathway, 33 key regulators in the auxin-signaling pathway; 16 key regulators in the GA-signaling pathway; and 21 key regulators in the cytokinin-signaling pathway were identified based on our transcriptome data (Figure 7). Most of these key regulators showed a flower stage-specific expression pattern, and the average expression values of these key regulators are listed in Table S6. Furthermore, seven key regulators were identified in both auxin- and ABA-signaling pathways, four key regulators were identified in both auxin- and GA-signaling pathways, and two key regulators were identified in both ABA- and cytokinin-signaling pathways. No crosstalk was found between GA and cytokinin during the flower development process in sugar apple.

### Validation of the expression of several key flowering-related genes in sugar apple

**Figure 8:**
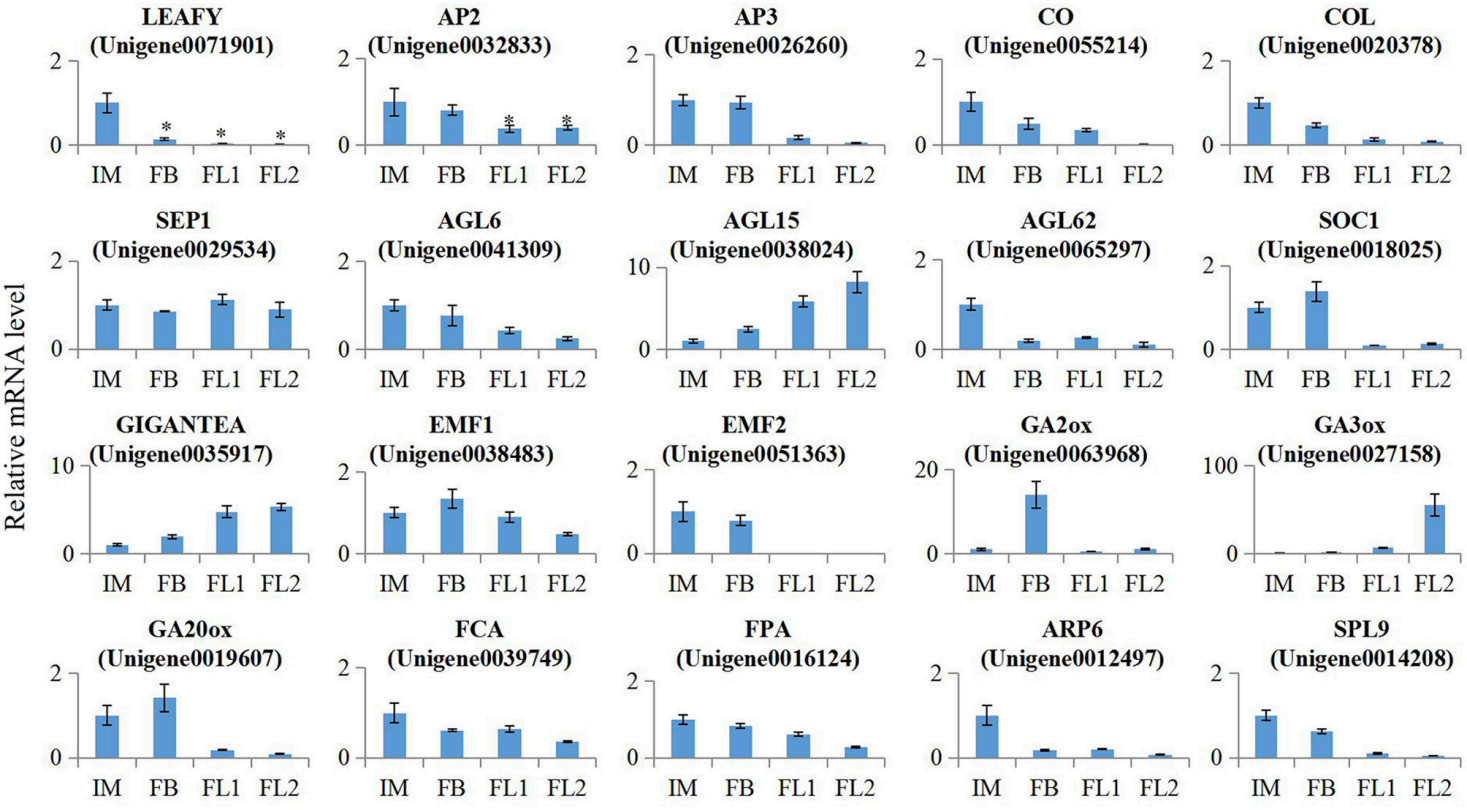
**Validation of the expression of flowering-related Genes in sugar apple**. Expression level of 20 flower development related genes in different stages of flowering process was validated by qRT-PCR. All these data were based on the analysis of three independent biological repeats. Significant differences in gene expressions were indicated by “^*^”.

To verify the expression of several key flowering-related genes that were identified using RNA-seq, we performed absolute quantification RT-PCR assays with independent samples collected from the flowers at different developmental stages (IM, FB, FL1, and FL2). We randomly selected 20 flowering-related unigenes to validate the RNA-seq data. The primer sequences and expression levels of these selected genes were listed in Table S7, S8. Next, we investigated whether changes in qRT-PCR correlated with the changes in RNA-seq data. Mostly, relatively high correlation coefficients were observed (*R*^2^ > 0.75). These results suggested that the expression levels of these selected genes were basically consistent with RNA-seq results (Figure 8, Table S9).

### Endogenous hormone measurements

**Figure 9:**
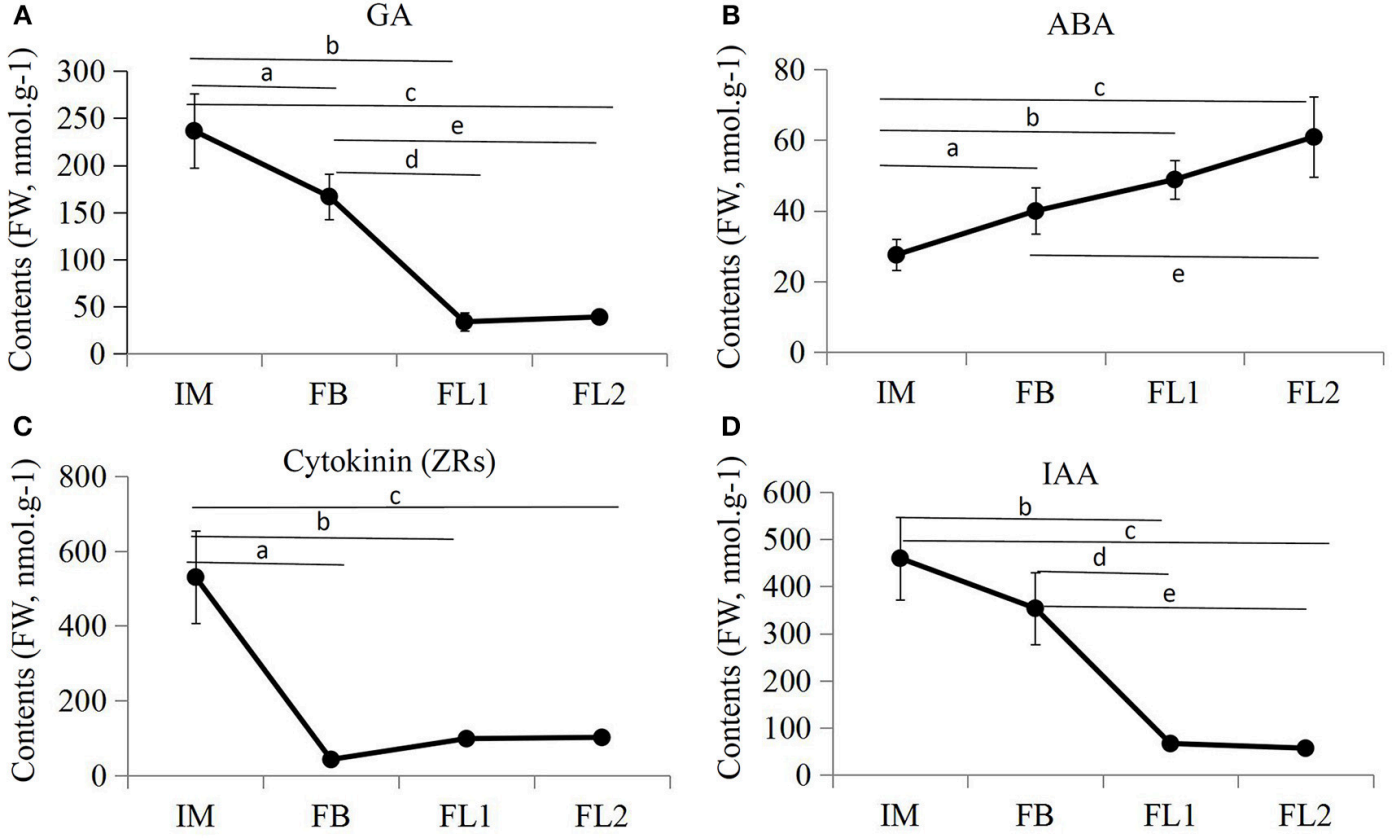
**Endogenous hormones measurements in various flowering stages**. The differences in endogenous **(A)** GA contents, **(B)** ABA contents, **(C)** ZA contents, and **(D)** IAA contents during four flower developmental stages in sugar apple were measured. The data were analyzed by three independent repeats, and standard deviations were shown with error bars. Significant differences in expression level between IM and FB were indicated by “a”; Significant differences in expression level between IM and FL1 were indicated by “b”; Significant differences in expression level between IM and FL2 were indicated by “c”; Significant differences in expression level between FB and FL1 were indicated by “d”; Significant differences in expression level between FB and FL2 were indicated by “e”.

To examine the roles of endogenous hormones during flower development, the contents of IAA, ABA, GA, and cytokinin were measured in sugar apple flowers at different stages. Independent samples collected from the flowers at different developmental stages (IM, FB, FL1, and FL2) were used for endogenous hormone measurements. The contents of IAA, GA, and ZRs decreased and the content of ABA increased during the flower development process (Figure 9). Both the GA and IAA contents reached their lowest points at stage FL1, while the ZRs content reached its lowest point at stage FL1. During the flowering process, the GA content decreased significantly from 236.55 nmol.g^−1^ FW (IM stage) to 38.71 nmol.g^−1^ FW (FL2 stage), the IAA content decreased significantly from 460.06 nmol.g^−1^ (IM stage) to 56.48 nmol.g^−1^ (FL2 stage), and the ZRs content decreased significantly from 530.66 nmol.g^−1^ (IM stage) to 56.48 nmol.g^−1^ (FL2 stage). On the contrary, the ABA content increased significantly from 27.52 nmol.g^−1^ (IM stage) to 101.32 nmol.g^−1^ (FL2 stage).

## Discussion

### Illumina sequencing and sequence annotation

The temporal regulation of gene expression plays an essential role in plant growth and development. Detailed information on gene expression is crucial for understanding the molecular mechanisms underlying any developmental process. Flower development, a key feature of higher plants, represents the reproductive phase of plant development (Krizek, 2015). Sugar apple is a popular and commercially important fruit tree in subtropical and tropical areas. However, little genomic information is available for sugar apple. An increasing number of studies have shown that Illumina sequencing is a powerful tool for DEG analyses during various flower developmental stages (Wong et al., 2013; Zhang et al., 2014b). Here, we describe a global view of gene expression dynamics during flower development in sugar apple, and it is the first attempt using Illumina sequencing technology to gain an insight into the transcriptome of sugar apple flowers. Our transcriptome data will meet the initial information needs for functional studies of this species and its relatives.

In this study, four RNA-seq experiments were performed using Illumina sequencing, which generated 71,948 unigenes. These unigenes were used for BLASTX-algorithm based searches and annotations against the protein databases Nr, SwissPort, COG, KEGG, and GO. In total, 24,911 unigenes (34.62%) were annotated in sugar apple, which is much smaller than other fruit trees, such as pineapple (58.33%), mango (85%), and loquat (70.57%), and similar to the number annotated in manchurian walnut (39.92%) and “Bartlett” pears (32.8%) (Gong et al., 2015; Nham et al., 2015; Sherman et al., 2015; Hu et al., 2016; Liu and Fan, 2016). This indicated that the functions of a large portion of the genes of sugar apple have not yet been identified.

Furthermore, cluster analysis of DEGs during the flower development has been performed by the K-means methods. Several stage-preferential expressed clusters have been identified in our study. Interstingly, a largest number of 8039 unigenes, which grouped into cluster 8, 9, 13, and 20, showed IM-preferential expression, suggesting the importance of IM stage in floral transition and flower development.

### Identification of TF genes in sugar apple flower development

Several molecular genetic studies have demonstrated the crucial roles of TFs in the reproductive development of plants (Smaczniak et al., 2012). Based on the GO analysis, the biological “regulation of transcription” was significantly enriched in DEGs during the flowering process. Among these TF families, MYB, bHLH, MADS-box, NAC, WRKY, bZIP, ARF, ERF, C2H2, and CCHC were found to be particularly important during flower development in *A. squamosa*. Some members of these families are also involved in reproductive development in other plant species (Sharma et al., 2012; Singh et al., 2013).

MYB TFs contain DNA-binding domains, and some of them have been identified as floral developmental regulators (Vimolmangkang et al., 2013). In *Arabidopsis*, several MYBs were reported to be involved in jasmonate-mediated stamen maturation. Transcriptional profiling indicated that MYB108 and MYB24 have overlapping functions and act downstream of MYB21, a R2R3-MYB TF, in a transcriptional cascade that mediates stamen and pollen maturation (Mandaokar and Browse, 2009; Song et al., 2011). Interestingly, the homologs of MYB24 and R2R3-MYB genes were also found in sugar apple. The increasing expression of MYB genes during the flowering process suggested an important regulatory role of MYBs in sugar apple flower development (Figure 4).

The bHLH consists of genes regulating various flower developmental processes, such as regulating the development of carpel margins, and controlling the morphogenesis of sepals, petals, stamens, and anthers in *A. thaliana* (Zhang et al., 2006). In sugar apple, a large number of bHLH homologs (717) were identified, and the average expression levels of these genes was induced during the flowering process. NACs have also been implicated in floral and vegetative development (Wellmer et al., 2004). In total, 457 NACs were identified in sugar apple and showed their highest expression levels at the FL2 flower developmental stage (Figure 4). In plants, the bZIP TFs function as key regulators of flower development, and light and stress responses (Running and Meyerowitz, 1996; Uno et al., 2000). In our study, 296 bZIPs were identified, and most of them showed differential expression patterns during the flowering process in sugar apple (Figure 4).

### Identification of the genes associated with floral transition and flower development

In *Arabidopsis*, the flowering signals arise mainly from six major flowering pathways (photoperiod, autonomous, vernalization, thermosensory, aging, and GA-induced) (Blázquez et al., 2003; Srikanth and Schmid, 2011). The photoperiod pathway is an important genetic network of flowering control (Komeda, 2004). The photoperiod pathway is comprised of three major parts including a circadian clock and an output pathway from the clock to flowering (Simpson, 2003). Light signals are first received by two photoreceptors, phytochromes and cryptochromes, and then they produce a circadian clock, which enables them to coordinate internal biological events with external rhythm changes (Imaizumi, 2010; Digel et al., 2015). A large number of flowering time-associated genes were identified in the model plant *Arabidopsis*. The circadian clock is composed of at least three interlocking loops to measure day length changes and regulate FKF1, GI, and CYCLING DOF FACTOR (CDF) (Imaizumi, 2010). FKF1 and GI form a complex to facilitate the expression of *CO*, which is a TF that promotes flowering by inducing the expression of the direct downstream genes, such as *FT* and *SOC1* (Kardailsky et al., 1999; Liu et al., 2008). The expression levels of both *CDF1* and *FT* in sugar apple were highest in the flower stage FB, suggesting a putative role in the early stage of flowering (Figure 5D). Interestingly, most of the photoperiod pathway-related genes showed lowest expression in the IM stage, suggesting that expression inhibition of these genes may play a role in the process of transition from vegetative to reproductive growth.

Woody plants undergo a long vegetative period to achieve transition to the reproductive stage (Rottmann et al., 2000; Huang et al., 2013). After this transition, woody plants start to form flower buds in the spring (Hsu et al., 2011). To date, studies are increasingly revealing the relationships between phytohormones and flowering. For example, cytokinins are believed to promote floral transition by activating MADS box family genes in *Sinapis alba* (Bonhomme et al., 2000). In our study, many CK signaling related genes and MADS-box genes were identified as differential expressed genes in sugar apple (Figures 7, 5H), suggesting an involvement of CK-MADS pathway in the regulation of floral transition. In classical flowering network, ABC model genes *AP* and *AG* genes are possibly activated by some floral integrators and consequently floral development (Huang et al., 2013). *AP1* and *AP2* genes belong to “class A” and specify sepal identify. *AG* genes belonged to “class C” is essential for carpe initiation (Bowman et al., 2012). In sugar apple, ABC model homologous genes *AP2* (Unigenes0032833 and Unigenes0033801), *AP3* (Unigene0026260) and *AG* (Unigene0058879) were identified as differential expressed genes (Figures 5E,H).

In this study, we have also detected 147 homologs of flowering- and flower development- related genes, based on sequence annotations and analyses of changes in gene expression during floral initiation and floral differentiation of sugar apple. Some of these genes encode regulators involved in flowering integrator or floral meristem identification, while others are related to flower development (Huang et al., 2013). These flowering-related genes were identified in our study, suggesting that all six of the flowering pathways may also be present in sugar apple (Table S4).

### The putative roles of phytohormone crosstalk in sugar apple flower development

A KEGG pathway enrichment analysis indicated that the DEGs were significantly enriched in the pathways related to phytohormone metabolism and signal transduction processes. Generally, many unigenes were annotated as genes related to hormone biosynthesis, transport, or signal transduction during flower development.

Auxin plays a pivotal role in plant flower development, including the initiation of floral primordia and the identification of floral organs (Alabadí et al., 2009). The expression levels of genes involved in auxin transport or signaling were altered in buds during floral initiation in litchi (Zhang et al., 2014b). The expression levels of many auxin-related genes were also significantly changed during floral differentiation in sugar apple. Several known ARFs and Aux/IAAs were identified, suggesting that the ARF-Aux/IAA regulatory pathway is vital for flower development in sugar apple. In the model plant *Arabidopsis, AtIAA10*, an earlier specifying regulator, was identified as a component of the auxin response machinery (Rademacher et al., 2012). In sugar apple, *IAA10* showed highest expression during the flower development process (Figure 7). It suggested a putative function of *IAA10* in earlier inflorescent meristem specifying. *Sl-IAA3*, an *Aux/IAA* gene in tomato, is a molecular link between auxin and ethylene responses (Salma et al., 2009). In sugar apple, the expression of *IAA3* was increased during floral transition and flower development, especially highest in the FL2 stage, which is not consistent with the change of IAA content, suggesting that *IAA3* may be regulated by ethylene during flower development. As a classical auxin response family, several *ARF* genes were involved in floral transition and flower development. In *Arabidopsis, ARF3* integrates the functions of *AGAMOUS* and *APETALA2* in floral meristem determinacy (Liu et al., 2014b). Another two members, *ARF6* and *ARF8* have conserved roles in controlling growth and development of vegetative and flower organs (Liu et al., 2014a). In our study, *ARF1* predominantly expressed in IM stage and *ARF19* highly expressed in FL1 stage, suggesting their different functions in flower development of sugar apple.

Moreover, increasing evidences showed that some auxin transporter genes were also involved in flower development. For example, *AtPIN1*-medicated auxin flux might regulate the early stages of female gametophyte development in *Arabidopsis* (Ceccato et al., 2013). The expression of *AtPIN1* is under control of *AtMLO4*, which encodes a heptahelical, plasma membrane-localized protein. An auxin transporter gene (*PIN7*) and a homologs of *AtMLO4* (MLO4) were identified in our study. Interestingly, both of *PIN7* and *MLO4* were decreased during flower development process in sugar apple. The decline in expression of *PIN7* may provide a partial explanation for the decreased IAA content during the flowering process in sugar apple (Figure 9D). *AVP1* encodes a proton pump that utilizes the energy released by hydrolysis of a pyrophosphate into two molecules of phosphate to acidify the vacuole (Heinonen, 2009). In our study, the phosphate requirement during the flower development may cause the induced expression of *AVP1* in sugar apple.

In addition to auxin, GA, ABA, and cytokinin also play important roles in the promotion of flowering. The GA effect on flowering is genetically mediated by five DELLA proteins, GIBBERELLIC ACID INSENSITIVE (GAI), REPRESSOR OF ga1-3 (RGA), RGA-LIKE1 (RGL1), RGL2, and RGL3 (Porri et al., 2012). ABA promotes floral initiation and floral differentiation in some woody plants (Shan et al., 2012). Cytokinin promotes *Arabidopsis* flowering through the transcriptional activation of the FT paralog TSF (D'Aloia et al., 2011). The homologs of the key genes in various phytohormone pathways were identified in sugar apple (Figure 7). The transcriptional dynamics of the hormone response genes and the changes in the contents of GA, ZRs, and ABA confirmed the involvement of various hormones in the flowering process (Figures 9A–C). *GA2ox* family genes, encoding the 2-oxoglutarate-dependent dioxygenases that catalyze the later steps in the biosynthetic pathway of GA, have been identified in different plant species (Pearce et al., 2015). Interestingly, two GA biosynthesis genes, *GA2OX6* and *GA2OX7*, showed significantly reduced expression during flowering process (Figure 7). It was in agreement with the decrease in the contents of GA during the development of flower. However, the expression of *GA3OX1* and *KAO1*, another two GA biosynthetic genes, was induced in sugar apple. In *Arabidopsis, GA3OX1* and *KAO1* play roles in both the synthesis of bioactive GA and environmental stimuli responses (Mitchum et al., 2006; Regnault et al., 2014). It suggested a diversity of regulation mechanism in GA biosynthesis during floral transition and flower development. Due to complex regulatory mechanism, the corresponding express trend of the hormone-related genes is not very clear. Further experiments are needed to reveal their specific functions in sugar apple.

Furthermore, seven regulators, LHY1, MIF1, DRB1, MYB60, MYB93, ETR1, and ABA3, were involved in both auxin- and ABA-signaling pathways. Among these regulators, LHY1, MIF1, MYB60, and ETR1 were controlled by both auxin- and GA-signaling pathways, while two regulators, DRB1 and MIF1, were under the control of ABA- and cytokinin-signaling pathways (Figure 7). Our data indicated crosstalk between different hormones during the flowering process in sugar apple.

In summary, four independent cDNA libraries from sugar apple flowers at the IM, FB, FL1, and FL2 stages were constructed and sequenced. A large number of DEGs were identified in sugar apple during the flowering process. Flower developmental stage-specific expression patterns of flowering time-associated and flower development-related genes were characterized based on GO and KEGG, and some were validated by a qRT-PCR analysis. Furthermore, the expression levels of hormone-related genes were analyzed during the flowering process. The identification and analyses of these hormone-related genes will aid us in elucidating the regulatory mechanisms of hormones during the flowering process in woody fruit plants.

## Authors contribution

KL, SF, YP, JZ, and YC carried out the molecular studies, participated in the analysis and drafted the manuscript. SF and YP carried out the qRT-PCR analysis. HL performed the statistical analysis. KL and CY conceived of the study, and participated in its design. KL and CY acquired of funding and helped to draft the manuscript. All authors read and approved the final manuscript.

### Conflict of interest statement

The authors declare that the research was conducted in the absence of any commercial or financial relationships that could be construed as a potential conflict of interest.
