# Supplementary material for: Transcriptome Analysis and Identification of Genes Associated with Floral Transition and Flower Development in Sugar Apple (Annona squamosa L.)
Source: Front Plant Sci. 2016 Nov 9;7:1695. doi: 10.3389/fpls.2016.01695 (PMC5101194; doi:10.3389/fpls.2016.01695)
Supplement: Supplementary file 1 [file Table1.DOCX]

Table S1 Throughput and quality of RNA-seq of the reference library and the DGE libraries

| Libaries | Total reads | Total mapped reads (%) | Uniquely mapped reads (%) | Total Nucleotides (nt) | Q20 percentage | N  percentage | GC  percentage |
| --- | --- | --- | --- | --- | --- | --- | --- |
| Reference library | 107,197,488 | - | - | 13,399,686,000 | 95.86% | 0.00% | 48.32% |
| IM | 25,464,610 | 18513305 (72.70) | 18488206 (72.60) | 3165901500 | 96.24% | 0.00% | 47.98% |
| FB | 31,628,420 | 22068147  (69.77) | 22042401 (69.69) | 3926896250 | 96.31% | 0.00% | 48.41% |
| FL1 | 24,384,248 | 18835642 (77.25) | 18799818 (77.10) | 3041623250 | 96.51% | 0.00% | 47.65% |
| FL2 | 25,720,210 | 19385368 (75.37) | 19338871 (75.19) | 3209009000 | 96.54% | 0.00% | 49.14% |
