# Supplementary material for: Transcriptome Analysis and Identification of Genes Associated with Floral Transition and Flower Development in Sugar Apple (Annona squamosa L.)
Source: Front Plant Sci. 2016 Nov 9;7:1695. doi: 10.3389/fpls.2016.01695 (PMC5101194; doi:10.3389/fpls.2016.01695)
Supplement: Supplementary file 2 [file Table2.DOCX]

Table S2 Summary of the transcriptome assembly

| Assembly statistics | |
| --- | --- |
| Total number of unigenes | 71,948 |
| Mean length of unigenes (bp) | 825.40 |
| Sequences with E-value < 1 e^-5^ against nr | 24,791 |
