# Supplementary material for: Transcriptome Analysis and Identification of Genes Associated with Floral Transition and Flower Development in Sugar Apple (Annona squamosa L.)
Source: Front Plant Sci. 2016 Nov 9;7:1695. doi: 10.3389/fpls.2016.01695 (PMC5101194; doi:10.3389/fpls.2016.01695)
Supplement: Supplementary file 3 [file Table3.DOCX]

Table S3 The KEGG pathways represented by all of the assembled unigenes.

| **#** | **Pathway** | **All genes with pathway annotation (6837)** | **Pathway ID** |
| --- | --- | --- | --- |
| 1 | [Metabolic pathways](#gene1) | 1892 (27.67%) | ko01100 |
| 2 | [Biosynthesis of secondary metabolites](#gene2) | 948 (13.87%) | ko01110 |
| 3 | [Ribosome](#gene3) | 394 (5.76%) | ko03010 |
| 4 | [Protein processing in endoplasmic reticulum](#gene4) | 201 (2.94%) | ko04141 |
| 5 | [Starch and sucrose metabolism](#gene5) | 200 (2.93%) | ko00500 |
| 6 | [Oxidative phosphorylation](#gene6) | 192 (2.81%) | ko00190 |
| 7 | [Plant-pathogen interaction](#gene7) | 191 (2.79%) | ko04626 |
| 8 | [Purine metabolism](#gene8) | 187 (2.74%) | ko00230 |
| 9 | [Plant hormone signal transduction](#gene9) | 187 (2.74%) | ko04075 |
| 10 | [Spliceosome](#gene10) | 178 (2.6%) | ko03040 |
| 11 | [RNA transport](#gene11) | 171 (2.5%) | ko03013 |
| 12 | [Glycolysis / Gluconeogenesis](#gene12) | 154 (2.25%) | ko00010 |
| 13 | [Phenylpropanoid biosynthesis](#gene13) | 133 (1.95%) | ko00940 |
| 14 | [Ubiquitin mediated proteolysis](#gene14) | 127 (1.86%) | ko04120 |
| 15 | [Amino sugar and nucleotide sugar metabolism](#gene15) | 125 (1.83%) | ko00520 |
| 16 | [Pyrimidine metabolism](#gene16) | 122 (1.78%) | ko00240 |
| 17 | [Endocytosis](#gene17) | 114 (1.67%) | ko04144 |
| 18 | [Carbon fixation in photosynthetic organisms](#gene18) | 114 (1.67%) | ko00710 |
| 19 | [Ribosome biogenesis in eukaryotes](#gene19) | 113 (1.65%) | ko03008 |
| 20 | [mRNA surveillance pathway](#gene20) | 106 (1.55%) | ko03015 |
| 21 | [Peroxisome](#gene21) | 105 (1.54%) | ko04146 |
| 22 | [RNA degradation](#gene22) | 104 (1.52%) | ko03018 |
| 23 | [Phagosome](#gene23) | 102 (1.49%) | ko04145 |
| 24 | [Pyruvate metabolism](#gene24) | 100 (1.46%) | ko00620 |
| 25 | [Glycerophospholipid metabolism](#gene25) | 97 (1.42%) | ko00564 |
| 26 | [Photosynthesis](#gene26) | 93 (1.36%) | ko00195 |
| 27 | [Pentose and glucuronate interconversions](#gene27) | 85 (1.24%) | ko00040 |
| 28 | [Phenylalanine metabolism](#gene28) | 78 (1.14%) | ko00360 |
| 29 | [Arginine and proline metabolism](#gene29) | 77 (1.13%) | ko00330 |
| 30 | [Glycerolipid metabolism](#gene30) | 74 (1.08%) | ko00561 |
| 31 | [Nucleotide excision repair](#gene31) | 71 (1.04%) | ko03420 |
| 32 | [Inositol phosphate metabolism](#gene32) | 70 (1.02%) | ko00562 |
| 33 | [Glutathione metabolism](#gene33) | 70 (1.02%) | ko00480 |
| 34 | [Phosphatidylinositol signaling system](#gene34) | 69 (1.01%) | ko04070 |
| 35 | [Cysteine and methionine metabolism](#gene35) | 68 (0.99%) | ko00270 |
| 36 | [Porphyrin and chlorophyll metabolism](#gene36) | 64 (0.94%) | ko00860 |
| 37 | [Fructose and mannose metabolism](#gene37) | 64 (0.94%) | ko00051 |
| 38 | [Pentose phosphate pathway](#gene38) | 63 (0.92%) | ko00030 |
| 39 | [Aminoacyl-tRNA biosynthesis](#gene39) | 63 (0.92%) | ko00970 |
| 40 | [Fatty acid metabolism](#gene40) | 61 (0.89%) | ko00071 |
| 41 | [Glyoxylate and dicarboxylate metabolism](#gene41) | 59 (0.86%) | ko00630 |
| 42 | [Citrate cycle (TCA cycle)](#gene42) | 59 (0.86%) | ko00020 |
| 43 | [Nitrogen metabolism](#gene43) | 58 (0.85%) | ko00910 |
| 44 | [DNA replication](#gene44) | 56 (0.82%) | ko03030 |
| 45 | [Alanine, aspartate and glutamate metabolism](#gene45) | 56 (0.82%) | ko00250 |
| 46 | [Valine, leucine and isoleucine degradation](#gene46) | 56 (0.82%) | ko00280 |
| 47 | [Basal transcription factors](#gene47) | 53 (0.78%) | ko03022 |
| 48 | [Galactose metabolism](#gene48) | 53 (0.78%) | ko00052 |
| 49 | [Terpenoid backbone biosynthesis](#gene49) | 52 (0.76%) | ko00900 |
| 50 | [alpha-Linolenic acid metabolism](#gene50) | 49 (0.72%) | ko00592 |
| 51 | [Proteasome](#gene51) | 48 (0.7%) | ko03050 |
| 52 | [RNA polymerase](#gene52) | 48 (0.7%) | ko03020 |
| 53 | [N-Glycan biosynthesis](#gene53) | 47 (0.69%) | ko00510 |
| 54 | [Propanoate metabolism](#gene54) | 47 (0.69%) | ko00640 |
| 55 | [Fatty acid biosynthesis](#gene55) | 46 (0.67%) | ko00061 |
| 56 | [Glycine, serine and threonine metabolism](#gene56) | 46 (0.67%) | ko00260 |
| 57 | [Homologous recombination](#gene57) | 45 (0.66%) | ko03440 |
| 58 | [Cyanoamino acid metabolism](#gene58) | 45 (0.66%) | ko00460 |
| 59 | [Protein export](#gene59) | 44 (0.64%) | ko03060 |
| 60 | [Ascorbate and aldarate metabolism](#gene60) | 44 (0.64%) | ko00053 |
| 61 | [Carotenoid biosynthesis](#gene61) | 42 (0.61%) | ko00906 |
| 62 | [Tyrosine metabolism](#gene62) | 42 (0.61%) | ko00350 |
| 63 | [Mismatch repair](#gene63) | 41 (0.6%) | ko03430 |
| 64 | [beta-Alanine metabolism](#gene64) | 41 (0.6%) | ko00410 |
| 65 | [Base excision repair](#gene65) | 41 (0.6%) | ko03410 |
| 66 | [Biosynthesis of unsaturated fatty acids](#gene66) | 40 (0.59%) | ko01040 |
| 67 | [Phenylalanine, tyrosine and tryptophan biosynthesis](#gene67) | 39 (0.57%) | ko00400 |
| 68 | [Flavonoid biosynthesis](#gene68) | 38 (0.56%) | ko00941 |
| 69 | [SNARE interactions in vesicular transport](#gene69) | 37 (0.54%) | ko04130 |
| 70 | [Stilbenoid, diarylheptanoid and gingerol biosynthesis](#gene70) | 36 (0.53%) | ko00945 |
| 71 | [Ubiquinone and other terpenoid-quinone biosynthesis](#gene71) | 35 (0.51%) | ko00130 |
| 72 | [Limonene and pinene degradation](#gene72) | 34 (0.5%) | ko00903 |
| 73 | [Tryptophan metabolism](#gene73) | 33 (0.48%) | ko00380 |
| 74 | [Circadian rhythm - plant](#gene74) | 33 (0.48%) | ko04712 |
| 75 | [Ether lipid metabolism](#gene75) | 29 (0.42%) | ko00565 |
| 76 | [Pantothenate and CoA biosynthesis](#gene76) | 28 (0.41%) | ko00770 |
| 77 | [Histidine metabolism](#gene77) | 28 (0.41%) | ko00340 |
| 78 | [Photosynthesis - antenna proteins](#gene78) | 27 (0.39%) | ko00196 |
| 79 | [Sphingolipid metabolism](#gene79) | 26 (0.38%) | ko00600 |
| 80 | [Valine, leucine and isoleucine biosynthesis](#gene80) | 25 (0.37%) | ko00290 |
| 81 | [Sulfur metabolism](#gene81) | 25 (0.37%) | ko00920 |
| 82 | [Caffeine metabolism](#gene82) | 25 (0.37%) | ko00232 |
| 83 | [Glycosylphosphatidylinositol(GPI)-anchor biosynthesis](#gene83) | 24 (0.35%) | ko00563 |
| 84 | [One carbon pool by folate](#gene84) | 24 (0.35%) | ko00670 |
| 85 | [Natural killer cell mediated cytotoxicity](#gene85) | 21 (0.31%) | ko04650 |
| 86 | [Lysine degradation](#gene86) | 21 (0.31%) | ko00310 |
| 87 | [Selenocompound metabolism](#gene87) | 21 (0.31%) | ko00450 |
| 88 | [Glycosaminoglycan degradation](#gene88) | 19 (0.28%) | ko00531 |
| 89 | [Other glycan degradation](#gene89) | 19 (0.28%) | ko00511 |
| 90 | [Butanoate metabolism](#gene90) | 19 (0.28%) | ko00650 |
| 91 | [Glycosphingolipid biosynthesis - globo series](#gene91) | 19 (0.28%) | ko00603 |
| 92 | [Regulation of autophagy](#gene92) | 19 (0.28%) | ko04140 |
| 93 | [Zeatin biosynthesis](#gene93) | 17 (0.25%) | ko00908 |
| 94 | [Folate biosynthesis](#gene94) | 17 (0.25%) | ko00790 |
| 95 | [Nicotinate and nicotinamide metabolism](#gene95) | 17 (0.25%) | ko00760 |
| 96 | [Steroid biosynthesis](#gene96) | 17 (0.25%) | ko00100 |
| 97 | [Isoquinoline alkaloid biosynthesis](#gene97) | 16 (0.23%) | ko00950 |
| 98 | [Flavone and flavonol biosynthesis](#gene98) | 15 (0.22%) | ko00944 |
| 99 | [Linoleic acid metabolism](#gene99) | 14 (0.2%) | ko00591 |
| 100 | [Taurine and hypotaurine metabolism](#gene100) | 14 (0.2%) | ko00430 |
| 101 | [Tropane, piperidine and pyridine alkaloid biosynthesis](#gene101) | 13 (0.19%) | ko00960 |
| 102 | [Lysine biosynthesis](#gene102) | 13 (0.19%) | ko00300 |
| 103 | [Diterpenoid biosynthesis](#gene103) | 13 (0.19%) | ko00904 |
| 104 | [Circadian rhythm - mammal](#gene104) | 12 (0.18%) | ko04710 |
| 105 | [Arachidonic acid metabolism](#gene105) | 12 (0.18%) | ko00590 |
| 106 | [Riboflavin metabolism](#gene106) | 11 (0.16%) | ko00740 |
| 107 | [Brassinosteroid biosynthesis](#gene107) | 10 (0.15%) | ko00905 |
| 108 | [Sulfur relay system](#gene108) | 10 (0.15%) | ko04122 |
| 109 | [Glucosinolate biosynthesis](#gene109) | 9 (0.13%) | ko00966 |
| 110 | [Thiamine metabolism](#gene110) | 9 (0.13%) | ko00730 |
| 111 | [Non-homologous end-joining](#gene111) | 9 (0.13%) | ko03450 |
| 112 | [Vitamin B6 metabolism](#gene112) | 9 (0.13%) | ko00750 |
| 113 | [Glycosphingolipid biosynthesis - ganglio series](#gene113) | 8 (0.12%) | ko00604 |
| 114 | [Fatty acid elongation in mitochondria](#gene114) | 8 (0.12%) | ko00062 |
| 115 | [C5-Branched dibasic acid metabolism](#gene115) | 6 (0.09%) | ko00660 |
| 116 | [ABC transporters](#gene116) | 4 (0.06%) | ko02010 |
| 117 | [Lipoic acid metabolism](#gene117) | 4 (0.06%) | ko00785 |
| 118 | [Other types of O-glycan biosynthesis](#gene118) | 4 (0.06%) | ko00514 |
| 119 | [Synthesis and degradation of ketone bodies](#gene119) | 4 (0.06%) | ko00072 |
| 120 | [Sesquiterpenoid biosynthesis](#gene120) | 3 (0.04%) | ko00909 |
| 121 | [Biotin metabolism](#gene121) | 3 (0.04%) | ko00780 |
| 122 | [Anthocyanin biosynthesis](#gene122) | 2 (0.03%) | ko00942 |
| 123 | [Betalain biosynthesis](#gene123) | 2 (0.03%) | ko00965 |
| 124 | [Monoterpenoid biosynthesis](#gene124) | 1 (0.01%) | ko00902 |
