# Supplementary material for: Transcriptome Analysis and Identification of Genes Associated with Floral Transition and Flower Development in Sugar Apple (Annona squamosa L.)
Source: Front Plant Sci. 2016 Nov 9;7:1695. doi: 10.3389/fpls.2016.01695 (PMC5101194; doi:10.3389/fpls.2016.01695)
Supplement: Supplementary file 4 [file Table4.DOCX]

Table S4 Genes that share homology to flowering genes of other plants.

| **UnigeneID** | **Unigene Length** | **Phase1(RPKM)** | **Phase2(RPKM)** | **Phase 3(RPKM)** | **Phase4(RPKM)** | **Annotation** |
| --- | --- | --- | --- | --- | --- | --- |
| **Photoperiod pathway** | |  |  |  |  |  |
| Unigene0035917 | 6214 | 40.1529478 | 66.6416 | 161.6475 | 172.237 | GIGANTEA |
| Unigene0035918 | 1652 | 0.425636181 | 0.52178 | 3.187656 | 4.06914 | GIGANTEA |
| Unigene0035919 | 1387 | 0.155987127 | 0.68688 | 2.301024 | 2.23689 | GIGANTEA |
| Unigene0039699 | 4631 | 8.537836317 | 27.7924 | 14.24273 | 37.8301 | Cryptochrome |
| Unigene0004635 | 2892 | 9.688057319 | 9.27108 | 6.878911 | 15.0014 | Cryptochrome |
| Unigene0015448 | 1790 | 9.760110168 | 4.02982 | 3.714525 | 19.4704 | Cryptochrome |
| Unigene0021309 | 1078 | 4.61609029 | 6.06017 | 26.79338 | 27.9652 | Phytochrome C |
| Unigene0065753 | 709 | 6.331944297 | 42.2957 | 13.80441 | 7.58501 | ELF1 |
| Unigene0033369 | 3703 | 0.146066801 | 0.47781 | 44.77437 | 177.247 | ELF3 |
| Unigene0032298 | 3419 | 7.656873803 | 7.97474 | 21.45416 | 48.7904 | ELF3 |
| Unigene0032296 | 568 | 23.71134775 | 24.7602 | 33.80689 | 59.1744 | ELF3 |
| Unigene0013296 | 2196 | 16.30537841 | 38.9422 | 28.89711 | 10.2901 | ELF4 |
| Unigene0042290 | 1514 | 0.750237292 | 0.92892 | 1.159403 | 0.85385 | ELF8 |
| Unigene0024687 | 3009 | 10.65952209 | 3.39236 | 7.530673 | 13.9713 | LHY-like |
| Unigene0056367 | 2739 | 7.681796492 | 1.68947 | 7.457368 | 10.6477 | LHY-like |
| Unigene0056365 | 592 | 1.46185233 | 2.22238 | 0.089851 | - | CHS |
| Unigene0048721 | 528 | 1.331725324 | 0.7733 | - | - | CHS |
| Unigene0055216 | 1180 | 0.366701941 | 372.164 | 0.090156 | 0.17529 | CHS |
| Unigene0036448 | 1592 | 633.3658871 | 218.828 | 618.3571 | 613.464 | CHS |
| Unigene0036447 | 1491 | 609.0855287 | 294.871 | 142.2378 | 123.638 | CHS |
| Unigene0057145 | 1443 | 4.760390922 | 27.8553 | 12.09077 | 5.33936 | CHS |
| Unigene0036300 | 1214 | 9.445444547 | 9.79092 | 7.010478 | 5.36687 | COP1-like |
| Unigene0055624 | 1358 | 0.398295554 | 0.56792 | 2.115146 | 4.18853 | COP1-like |
| Unigene0063214 | 1638 | 14.52927713 | 6.03787 | 8.443175 | 4.04078 | CO |
| Unigene0055214 | 1560 | 2.357705425 | 1.48316 | 1.022923 | 0.06629 | CO |
| Unigene0020378 | 854 | 21.40740661 | 12.6964 | 4.484572 | 2.72473 | COL |
| Unigene0031390 | 2991 | 3.074239772 | 9.07039 | 8.963146 | 8.69602 | COL |
| Unigene0047636 | 809 | 0.66858512 | 0.28039 | 1.052005 | 0.83093 | COL |
| Unigene0060558 | 548 | 0.493508542 | 4.88441 | 0.194131 | 3.68004 | COL |
| Unigene0019116 | 2250 | 0.048078699 | 0.14114 | 3.404288 | 17.788 | COL |
| Unigene0023936 | 1590 | 151.6520091 | 82.7736 | 28.03453 | 11.0248 | COL |
| Unigene0037475 | 2427 | 47.17982332 | 37.0676 | 40.43644 | 16.299 | COL |
| Unigene0011512 | 1725 | 25.74300768 | 10.2306 | 1.048422 | 1.07915 | COL |
| Unigene0012610 | 1652 | 18.466062 | 23.3426 | 5.538151 | 2.8797 | COL |
| Unigene0023935 | 1493 | 14.99842867 | 3.06904 | 2.030773 | 1.80099 | COL |
| **other flowering genes** | |  |  |  |  |  |
| Unigene0053291 | 1424 | 1.595307951 | 2.26198 | 11.16883 | 2.39664 | GA2ox |
| Unigene0063968 | 1265 | 1.026185668 | 44.1836 | 1.219421 | 2.37086 | GA2ox |
| Unigene0034393 | 1332 | - | - | 11.58084 | 18.4011 | GA2ox |
| Unigene0027158 | 1721 | 0.31428551 | 0.55358 | 4.79068 | 76.7377 | GA3ox |
| Unigene0069334 | 1970 | 10.32349727 | 13.2647 | 22.41084 | 10.4206 | GA20ox |
| Unigene0019607 | 1753 | 7.775419925 | 14.4409 | 1.33511 | 1.06191 | GA20ox |
| Unigene0020705 | 1736 | 5.764043319 | 6.89915 | 3.554304 | 1.90633 | GA20ox |
| Unigene0006367 | 2516 | 1.526345816 | 3.44401 | 78.98463 | 127.403 | GA20ox |
| Unigene0037073 | 676 | 4.480707143 | 6.91244 | 0.314746 | 10.0206 | GA20ox |
| Unigene0055088 | 1039 | 4.37289417 | 1.13527 | - | 0.09954 | GID1 |
| Unigene0071035 | 408 | 0.397709825 | 1.44552 | 0.391118 | 0.38022 | GAI |
| Unigene0008226 | 693 | 18.26366158 | 16.4317 | 60.33033 | 15.7441 | RGL3 |
| **Vernalization pathway** | |  |  |  |  |  |
| Unigene0038483 | 4468 | 5.94392822 | 7.99103 | 5.643019 | 3.57614 | EMF1 |
| Unigene0039768 | 580 | 8.393048725 | 4.38027 | 2.201048 | 0.62408 | EMF2 |
| Unigene0051363 | 355 | 0.914172443 | 0.63897 | 0.149837 | - | EMF2 |
| Unigene0039058 | 245 | 1.103847678 | 0.55552 | 0.651331 | - | EMF2 |
| Unigene0043201 | 450 | 0.360590242 | 0.80653 | 0.236409 | - | EMF2 |
| Unigene0022903 | 465 | 0.232638865 | 1.46346 | 0.228783 | 0.22241 | EMF2 |
| Unigene0066091 | 222 | 0.243642055 | 0.20436 | - | - | EMF2 |
| Unigene0035539 | 2741 | 21.5091224 | 13.3403 | 14.34108 | 11.9039 | EMF2 |
| Unigene0026525 | 2814 | 14.53124854 | 12.4945 | 9.3379 | 8.21395 | EMF2 |
| Unigene0031484 | 1691 | 27.50806692 | 41.8257 | 45.20219 | 27.4906 | FIE |
| Unigene0026747 | 2387 | 14.13960897 | 16.4211 | 14.19493 | 9.68331 | VIN3 |
| Unigene0026749 | 582 | 4.089167687 | 5.22267 | 4.75255 | 2.31004 | VIN3 |
| Unigene0026748 | 3362 | 89.8365218 | 72.5577 | 52.21107 | 53.5242 | VIN3 |
| **Autonomous pathway** | |  |  |  |  |  |
| Unigene0012503 | 2702 | 397.4166209 | 623.873 | 229.58 | 141.732 | FRI |
| Unigene0012518 | 2449 | 87.88006764 | 118.114 | 70.69823 | 46.9586 | FRI |
| Unigene0032976 | 2695 | 47.66614974 | 38.0444 | 37.8956 | 15.8102 | FRI |
| Unigene0006320 | 2425 | 8.408815735 | 4.41511 | 6.887542 | 3.85954 | FRI |
| Unigene0034535 | 2875 | 5.512327344 | 3.21909 | 5.698483 | 3.65113 | FRI |
| Unigene0025842 | 5206 | 9.755884656 | 5.49878 | 8.163736 | 7.37995 | LD |
| Unigene0039749 | 2568 | 33.67896006 | 20.2279 | 25.80889 | 16.4511 | FCA |
| Unigene0025427 | 4007 | 17.8315339 | 11.5597 | 18.70415 | 11.0723 | FPA |
| Unigene0016124 | 1753 | 15.42742049 | 13.4057 | 12.80492 | 6.28299 | FPA |
| Unigene0016122 | 1676 | 5.809031337 | 6.95665 | 8.124793 | 6.0163 | FPA |
| Unigene0041654 | 3925 | 12.30600327 | 6.35717 | 9.879483 | 3.37263 | FY |
| Unigene0039190 | 2646 | 14.98371015 | 18.4143 | 8.26225 | 5.78457 | DCL |
| Unigene0039696 | 3955 | 9.750980109 | 7.91487 | 5.43352 | 3.8831 | DCL |
| Unigene0036202 | 1889 | 16.77918593 | 9.70265 | 7.490245 | 2.38153 | DCL |
| Unigene0039188 | 1510 | 13.86242617 | 8.02187 | 10.39182 | 4.31482 | DCL |
| Unigene0039187 | 3077 | 5.519597132 | 8.07968 | 5.791135 | 3.66351 | DCL |
| Unigene0039694 | 1797 | 3.009935238 | 2.92854 | 4.144063 | 1.66897 | DCL |
| Unigene0039182 | 293 | 1.846025127 | 0.77418 | 1.996969 | 1.05889 | DCL |
| **Thermosensory pathway** | | |  |  |  |  |
| Unigene0012497 | 1611 | 7.755711898 | 1.23908 | 1.81599 | 0.89873 | ARP6 |
| Unigene0028128 | 3788 | 10.18086756 | 7.10208 | 6.670064 | 3.34445 | HOS1 |
| **Aging pathway** |  |  |  |  |  |  |
| Unigene0014208 | 1606 | 18.65818747 | 14.0113 | 1.755402 | 1.19131 | SPL9 |
| Unigene0016634 | 891 | 13.35519413 | 7.07747 | 1.671578 | 0.63839 | SPL9 |
| Unigene0032833 | 1959 | 45.36368812 | 42.2407 | 17.10616 | 24.5745 | AP2 |
| Unigene0033801 | 2244 | 19.57214412 | 4.71058 | 14.19876 | 4.70085 | AP2 |
| **Floral pathway integrator or identity genes** | | | |  |  |  |
| Unigene0007110 | 587 | 0.737152112 | 1.39115 | 1.359251 | 0.52855 | FT |
| Unigene0018024 | 350 | 4.327082898 | 3.37013 | 0.303954 | 1.32967 | SOC1 |
| Unigene0018025 | 639 | 4.147634233 | 5.25378 | 0.249728 | 0.80922 | SOC1 |
| Unigene0071901 | 1523 | 7.564581889 | 1.01279 | 0.209555 | 0.13581 | LEAFY |
| **Other flowering genes** | |  |  |  |  |  |
| Unigene0040247 | 3044 | 104.5877543 | 98.6332 | 45.67802 | 26.2114 | AP3 |
| Unigene0026260 | 1319 | 196.1785878 | 189.62 | 26.77748 | 8.58555 | AP3 |
| Unigene0029534 | 1521 | 66.89055664 | 54.9715 | 84.21193 | 61.6364 | SEP1 |
| Unigene0058879 | 835 | 1.295533802 | 1.30396 | 0.127406 | 0.30964 | AG |
| Unigene0041309 | 1057 | 71.23107136 | 59.7884 | 31.75417 | 21.7209 | AGL6 |
| Unigene0038024 | 3058 | 2.01638101 | 3.1303 | 8.192751 | 10.1964 | AGL15 |
| Unigene0037345 | 545 | 0.893205185 | 0.99891 | 1.464 | 0.94879 | AGL16 |
| Unigene0006188 | 461 | 0.58664356 | 1.18092 | 0.807688 | - | AGL16 |
| Unigene0024611 | 1045 | 6.521679966 | 5.55693 | 2.494171 | 0.74224 | AGL26 |
| Unigene0065297 | 654 | 3.308167353 | 0.97116 | 1.708 | 0.63253 | AGL62 |
| Unigene0038230 | 1600 | 2.332568125 | 1.50279 | 3.9894 | 2.16533 | LEUNIG |
| Unigene0038222 | 962 | 0.393575627 | 0.04716 | 0.276466 | 0.1075 | LEUNIG |
| Unigene0038227 | 946 | 1.886809403 | 2.58967 | 4.104668 | 2.07712 | LEUNIG |
| Unigene0038226 | 1648 | 5.448238479 | 5.72595 | 11.00636 | 4.86344 | LEUNIG[Vitis vinifera] |
| Unigene0038225 | 371 | 15.5996587 | 13.6957 | 20.07245 | 11.0109 | LEUNIG-like |
| Unigene0038220 | 837 | 5.298996381 | 4.44457 | 4.956961 | 2.90363 | LEUNIG-like |
| Unigene0035194 | 2353 | 1.563119619 | 0.36633 | 1.288544 | 0.37359 | LEUNIG-like |
| Unigene0038218 | 534 | 2.430945448 | 2.12393 | 3.486367 | 1.74301 | LEUNIG-like |
| Unigene0038238 | 691 | 0.391378699 | 0.19696 | 0.384891 | 0.37416 | LEUNIG-like |
| Unigene0035181 | 205 | 0.791539555 | 0.44261 | 2.854205 | 0.50448 | LEUNIG-like |
| Unigene0035187 | 2656 | 66.87754253 | 76.8474 | 35.86855 | 21.3963 | LEUNIG-like |
| Unigene0035178 | 917 | 32.20538798 | 42.6461 | 30.1634 | 12.5185 | LEUNIG-like |
| Unigene0037747 | 566 | 19.68593368 | 22.3629 | 18.23189 | 9.04456 | LEUNIG-like |
| Unigene0038245 | 621 | 14.28425111 | 22.574 | 11.47782 | 6.57816 | LEUNIG-like |
| Unigene0022791 | 3057 | 64.63376606 | 12.4659 | 6.664226 | 8.06848 | ANT-like |
| Unigene0041141 | 2795 | 31.52423095 | 10.0635 | 6.794113 | 6.19772 | ANT-like |
| Unigene0041143 | 1308 | 25.0593677 | 7.21434 | 9.760001 | 4.62538 | ANT-like |
| Unigene0057742 | 530 | 0.306161526 | 0.59919 | - | 0.19513 | MADS-1 protein |
| Unigene0016742 | 1648 | 0.098462141 | - | 3.066287 | 2.41603 | MADS-2 protein |
| Unigene0041301 | 1392 | 3.613673757 | 8.14783 | 1.146379 | 0.74295 | MADS-box protein |
| Unigene0003721 | 1320 | 1.352213406 | 6.83944 | 29.3362 | 5.40598 | MADS-box protein |
| Unigene0005407 | 816 | 1.922264155 | 0.94515 | 1.173353 | 0.88717 | MADS-box protein |
| Unigene0058879 | 835 | 1.295533802 | 1.30396 | 0.127406 | 0.30964 | MADS-box protein |
| Unigene0002136 | 899 | 0.721982686 | 2.97737 | 0.828352 | 1.8406 | MADS-box protein |
| Unigene0056533 | 503 | 1.290382574 | 1.53328 | 0.951746 | 0.92522 | MADS-box protein |
| Unigene0038261 | 1671 | 86.4251297 | 69.6118 | 169.9534 | 203.619 | MADS-box protein |
| Unigene0005433 | 826 | 1.898992192 | 0.54924 | 4.4434 | 1.87806 | MADS-box protein 26 |
| Unigene0037346 | 2810 | 4.253938258 | 4.81117 | 3.520894 | 1.8954 | MADS-box protein 27 |
| Unigene0027497 | 1267 | 16.05153088 | 12.8904 | 22.88054 | 14.6108 | SPL1 |
| Unigene0070526 | 1468 | 53.79377581 | 34.6434 | 24.34947 | 6.86875 | SPL1 |
| Unigene0035811 | 3083 | 39.10585379 | 13.6852 | 8.55765 | 2.16364 | SPL6 |
| Unigene0031254 | 3957 | 9.718713484 | 9.92871 | 12.62252 | 12.4406 | SPL7 |
| Unigene0028203 | 3373 | 95.34848396 | 36.3421 | 20.01205 | 12.4483 | SPL8 |
| Unigene0028605 | 2567 | 20.2911026 | 23.3816 | 8.184979 | 3.74676 | SPL12 |
| Unigene0028604 | 3016 | 56.16886454 | 35.5446 | 7.813017 | 4.01193 | SPL12 |
| Unigene0027496 | 2898 | 29.8252177 | 30.3543 | 31.27646 | 18.8245 | SPL12 |
| Unigene0021954 | 3232 | 25.45441324 | 24.8312 | 27.78097 | 16.3192 | SPL14 |
| Unigene0023431 | 4617 | 58.65741843 | 53.7389 | 43.16882 | 57.018 | SPL14 |
| Unigene0021953 | 1189 | 59.00154036 | 52.2351 | 35.38677 | 16.0477 | SPL14 |
| Unigene0016633 | 1590 | 10.37547393 | 6.59107 | 2.241424 | 0.87808 | SPL17 |
| Unigene0036994 | 2197 | 12.94973603 | 1.87911 | 4.769606 | 3.1068 | CLV1 |
| Unigene0029893 | 1046 | 9.928297281 | 1.04093 | 2.034111 | 2.12572 | CLV1 |
| Unigene0027229 | 948 | 1.939884211 | 2.39278 | 1.402743 | 0.32727 | CLV2 |
| Unigene0032316 | 525 | 0.103025783 | 0.86414 | 1.013181 | 0.39398 | CLV2 |
| Unigene0032317 | 2632 | 7.932437304 | 5.82602 | 3.920687 | 2.63262 | CLV2 |
| Unigene0063540 | 1000 | 6.003827521 | 1.17954 | 0.957456 | 0.82735 | WUS |
| Unigene0016105 | 375 | 7.067568734 | 7.37972 | 3.687979 | 0.13789 | KNOTTED-1 |
| Unigene0023642 | 1677 | 35.47846741 | 7.65587 | 15.98615 | 11.0079 | KNOTTED-like |
| Unigene0022975 | 1982 | 19.56684181 | 4.23457 | 14.92167 | 15.4971 | KNOTTED-like |
