# Supplementary material for: Transcriptome Analysis and Identification of Genes Associated with Floral Transition and Flower Development in Sugar Apple (Annona squamosa L.)
Source: Front Plant Sci. 2016 Nov 9;7:1695. doi: 10.3389/fpls.2016.01695 (PMC5101194; doi:10.3389/fpls.2016.01695)
Supplement: Supplementary file 5 [file Table5.DOCX]

Table S5 The saw data of circadian rhythm related genes.

| GeneID | IM_RPKM | FB_RPKM | FL1_RPKM | FL2_RPKM |
| --- | --- | --- | --- | --- |
| ELF3 |  |  |  |  |
| Unigene0013296 | 16.30537841 | 38.94216745 | 28.89711345 | 10.29006184 |
| Unigene0032298 | 7.656873803 | 7.974738891 | 21.45416026 | 48.79037385 |
| Unigene0032296 | 23.71134775 | 24.76021772 | 33.80689006 | 59.17440564 |
| Unigene0065753 | 6.331944297 | 42.29571056 | 13.80441297 | 7.58500703 |
| Unigene0042290 | 0.750237292 | 0.928917019 | 1.159403 | 0.85385282 |
| Unigene0033369 | 0.146066801 | 0.477806437 | 44.77436603 | 177.2472279 |
| SPA1 |  |  |  |  |
| Unigene0039924 | 9.744808039 | 8.068880925 | 10.1558286 | 8.348763677 |
| Unigene0028848 | 9.706867826 | 7.807902888 | 3.777551169 | 2.43162861 |
| Unigene0025419 | 7.32432798 | 4.987394705 | 7.629314102 | 10.49582384 |
| Unigene0029636 | 4.511039577 | 11.84237549 | 13.29440021 | 32.61062065 |
| PRR |  |  |  |  |
| Unigene0021444 | 32.80892235 | 47.46185808 | 56.47234613 | 59.4117597 |
| Unigene0031256 | 106.3346817 | 86.01320246 | 110.4232764 | 107.6361955 |
| LHY |  |  |  |  |
| Unigene0024687 | 10.65952209 | 3.392356077 | 7.530672594 | 13.97131362 |
| Unigene0056367 | 7.681796492 | 1.689465161 | 7.457367526 | 10.64770365 |
| CRY |  |  |  |  |
| Unigene0039699 | 8.537836317 | 27.79237467 | 14.24273058 | 37.8301013 |
| Unigene0004635 | 9.688057319 | 9.271079247 | 6.878910654 | 15.00142641 |
| GI |  |  |  |  |
| Unigene0035917 | 40.1529478 | 66.64161045 | 161.6475393 | 172.2368274 |
| Unigene0035918 | 0.425636181 | 0.521776673 | 3.187656432 | 4.069135884 |
| Unigene0035919 | 0.155987127 | 0.686884845 | 2.301023979 | 2.23688508 |
| PAP1 |  |  |  |  |
| Unigene0020140 | 24.6071393 | 13.25405353 | 18.28475148 | 19.61647426 |
| FT |  |  |  |  |
| Unigene0007110 | 0.737152112 | 1.391154949 | 1.359250536 | 0.528545078 |
| CDF1 |  |  |  |  |
| Unigene0063951 | 82.84510437 | 230.5950148 | 89.54482661 | 56.18695004 |
| Unigene0024220 | 7.521746128 | 10.3986105 | 8.140495418 | 5.600940356 |
| CK2ɑ |  |  |  |  |
| Unigene0016225 | 106.1556805 | 205.5603106 | 119.6579627 | 55.29375871 |
| Unigene0022963 | 42.24996684 | 48.10136733 | 58.88662808 | 88.82030277 |
| Unigene0032829 | 20.65791082 | 24.56929556 | 21.75745236 | 19.50002323 |
| Unigene0022964 | 67.78040418 | 83.61594702 | 116.2879592 | 138.4684901 |
| Unigene0032839 | 21.86100719 | 30.90549801 | 36.92275175 | 25.52088918 |
| CK2β |  |  |  |  |
| Unigene0021099 | 75.61426547 | 141.3337807 | 94.11411662 | 88.47603441 |
| Unigene0038798 | 26.23399695 | 24.9982027 | 27.09258404 | 24.92286267 |
| Unigene0038799 | 24.89988042 | 22.91056447 | 17.5668744 | 11.50423051 |
| ZTL |  |  |  |  |
| Unigene0018901 | 9.416372996 | 9.463141456 | 9.880077565 | 8.080689859 |
| Unigene0018900 | 22.03607031 | 37.01246617 | 25.11844648 | 30.00417726 |
| PIF3 |  |  |  |  |
| Unigene0019635 | 13.14280908 | 6.345300847 | 5.926568635 | 2.819394348 |
| Unigene0011272 | 1.206331461 | 1.623615401 | 1.57258519 | 1.877413317 |
| Unigene0011271 | 2.527501693 | 2.75594585 | 1.408511017 | 0.724897104 |
| Unigene0011273 | 0.428141448 | 1.316723469 | 2.105224445 | 1.091489747 |
| TOC1 |  |  |  |  |
| Unigene0025643 | 3.408865173 | 1.627314992 | 10.7168604 | 9.759420374 |
| COP1 |  |  |  |  |
| Unigene0036300 | 9.445444547 | 9.790924591 | 7.010478327 | 5.366865876 |
| Unigene0055624 | 0.398295554 | 0.56792404 | 2.115145974 | 4.188531624 |
| CIP1 |  |  |  |  |
| Unigene0029721 | 22.04790494 | 17.05530399 | 15.11201411 | 6.998254752 |
| Unigene0020584 | 8.426632601 | 8.926617924 | 13.08978603 | 12.61696778 |
| CHE |  |  |  |  |
| Unigene0002541 | 21.33334937 | 21.41675895 | 21.16540485 | 13.53647298 |
| PHYA |  |  |  |  |
| Unigene0026313 | 9.239577806 | 14.84869169 | 52.32327592 | 43.49243971 |
| PHYB |  |  |  |  |
| Unigene0020702 | 9.889236801 | 10.8873407 | 38.21703051 | 50.49892288 |
