# Supplementary material for: Transcriptome Analysis and Identification of Genes Associated with Floral Transition and Flower Development in Sugar Apple (Annona squamosa L.)
Source: Front Plant Sci. 2016 Nov 9;7:1695. doi: 10.3389/fpls.2016.01695 (PMC5101194; doi:10.3389/fpls.2016.01695)
Supplement: Supplementary file 6 [file Table6.DOCX]

Table S6 The average expression values of key regulators in various hormones.

|  |  | IM_RPKM | FB_RPKM | FL1_RPKM | FL2_RPKM |
| --- | --- | --- | --- | --- | --- |
| ABA | LHY | 10.19513443 | 8.559177832 | 12.22586063 | 11.61654491 |
|  | PYL9 | 7.878001943 | 10.39017235 | 9.268819308 | 8.065765338 |
|  | MPK11 | 32.27170846 | 33.67356387 | 30.72314107 | 23.29985056 |
|  | ABCC5 | 4.818087935 | 5.045487492 | 25.01736376 | 18.96248134 |
|  | AAO4 | 8.848058096 | 11.87717258 | 14.78648878 | 11.86884582 |
|  | KAT1 | 20.40313042 | 54.51339215 | 117.9938698 | 366.1499174 |
|  | MYB13 | 11.03620842 | 11.99432304 | 27.91580836 | 43.98749264 |
|  | ASK9 | 7.539104338 | 6.342933496 | 5.377422998 | 4.323298383 |
|  | SMO2-2 | 48.12474144 | 15.15296136 | 11.68803871 | 3.74958749 |
|  | POL2A | 7.764870925 | 2.530034634 | 1.129262716 | 0.363373187 |
|  | MYB60 | 21.04802767 | 14.23770603 | 46.34959393 | 39.37864116 |
|  | DRB1 | 41.68636207 | 32.36236763 | 27.22247268 | 12.02285843 |
|  | ACR8 | 17.41906045 | 12.03544006 | 23.0505212 | 42.9825786 |
|  | SLAC1 | 0.207235771 | 1.354523021 | 1.716319182 | 2.781467961 |
|  | GAI | 15.41244358 | 7.932598538 | 17.27204127 | 6.684983172 |
|  | LPP2 | 38.65049153 | 34.28252396 | 35.5685468 | 36.79645774 |
|  | RHA2A | 31.59784181 | 39.32258901 | 10.12439321 | 10.8264037 |
|  | ADAP | 24.00682378 | 9.268170031 | 5.487314756 | 5.157626797 |
|  | ABA3 | 33.67646436 | 75.27467767 | 36.36028504 | 20.91820464 |
|  | ATMYB52 | 18.38193796 | 40.0600724 | 12.242576 | 7.091351202 |
|  | RACK1A | 1.34731669 | 0.319049642 | 0.360013566 | 0.542405526 |
|  | CPK10 | 21.25347742 | 21.59649921 | 11.67777867 | 12.86999433 |
|  | PUB44 | 7.224259531 | 9.624469537 | 6.102268126 | 7.851976611 |
|  | ZAT10 | 121.7316026 | 65.99642631 | 40.14250445 | 132.8535746 |
|  | LEC2 | 3.091721215 | 1.574627869 | 2.153233211 | 1.456123806 |
|  | NCED5 | 40.23131935 | 42.10751769 | 100.372135 | 59.36073885 |
|  | XLG3 | 15.62103959 | 15.49607215 | 12.47275631 | 9.448235714 |
|  | AtMYB93 | 2.504098899 | 1.575246826 | 0.12312964 | 0 |
|  | ANN1 | 54.52887126 | 77.12246024 | 106.9586394 | 65.90081743 |
|  | RAP2-6 | 70.06005825 | 32.98119765 | 43.98253621 | 30.09599843 |
|  | ALDH3H1 | 3.512232635 | 4.713085235 | 3.394414828 | 4.0307706 |
|  | ABF2 | 13.36388102 | 13.51087508 | 12.4732416 | 14.51841888 |
|  | LACS2 | 15.70039031 | 17.35702279 | 26.2375088 | 34.95741366 |
|  | ALATS | 21.78588765 | 18.91477237 | 20.47940233 | 14.71450913 |
|  | ABA2 | 32.73611342 | 107.9378393 | 108.2931029 | 88.67667196 |
|  | GCR2 | 12.42554275 | 20.08758237 | 10.56713604 | 5.03723428 |
|  | LOX1 | 0 | 0.360056417 | 0.211079382 | 0.410391482 |
|  | PARN | 9.829926545 | 6.521087431 | 8.543143438 | 3.789140468 |
|  | LOS1 | 2.462725542 | 1.473444398 | 2.580116294 | 1.528705016 |
|  | RBOHF | 0.645191287 | 1.623475059 | 0.845996092 | 0.308405527 |
|  | GTG1 | 10.08337453 | 13.88132401 | 12.12583685 | 5.789139421 |
|  | ETR1 | 10.76303785 | 18.42770109 | 5.241915775 | 7.513041793 |
|  | RGL1 | 15.92137062 | 8.69716154 | 8.316900741 | 6.327449911 |
|  | RPK1 | 36.93085742 | 34.57377329 | 16.15308448 | 13.2023813 |
|  | MIF1 | 66.75478914 | 17.09800437 | 73.56458101 | 128.1896225 |
|  | GASA1 | 29.89558073 | 123.1289417 | 3.058965104 | 6.938631708 |
| Auxin | NGA3 | 9.240689062 | 18.24946807 | 15.67008356 | 20.23726359 |
|  | LHY | 10.19513443 | 8.559177832 | 12.22586063 | 11.61654491 |
|  | IAA10 | 70.71806804 | 41.39882606 | 26.34961569 | 21.25565214 |
|  | IAA3 | 28.88998897 | 14.36321436 | 14.41531546 | 34.53205796 |
|  | IAA12 | 0.131923259 | 0.99586336 | 2.20552213 | 0.882842164 |
|  | AXR1 | 16.23839643 | 13.67962268 | 17.80826402 | 12.31066686 |
|  | MYB60 | 21.04802767 | 14.23770603 | 46.34959393 | 39.37864116 |
|  | MYB61 | 5.355087307 | 4.009423298 | 1.079741495 | 0.682317182 |
|  | DRB1 | 41.68636207 | 32.36236763 | 27.22247268 | 12.02285843 |
|  | SRK2A | 16.29202729 | 6.836467387 | 9.688265358 | 7.71833568 |
|  | MLO4 | 8.727608115 | 6.509437378 | 5.57215553 | 4.83774513 |
|  | MLO2 | 1.74272518 | 0.699346037 | 4.206438505 | 13.10558193 |
|  | AFB3 | 49.01305209 | 42.87825378 | 50.48168931 | 21.98530532 |
|  | IAA34 | 1.072577206 | 0.728272754 | 1.105027474 | 0.683598277 |
|  | AVP1 | 45.74408329 | 53.43587616 | 153.5359666 | 201.9286402 |
|  | TPL | 36.31590929 | 36.0834082 | 38.92543497 | 24.86361826 |
|  | SAUR41 | 4.830688627 | 9.29779023 | 3.708618152 | 2.387633822 |
|  | ABA3 | 33.67646436 | 75.27467767 | 36.36028504 | 20.91820464 |
|  | CHMP1A | 53.57846517 | 34.63161678 | 23.29244108 | 25.51868283 |
|  | MKK7 | 15.66077519 | 13.00397632 | 10.53733178 | 8.208072153 |
|  | ARF19 | 27.32595919 | 17.72983572 | 28.12665792 | 24.68429063 |
|  | CSN5B | 105.3031236 | 88.16545551 | 102.4888859 | 89.11170913 |
|  | PIN7 | 17.51371035 | 8.255290352 | 9.925824765 | 5.639774577 |
|  | PERK12 | 1.366278032 | 0.404461592 | 0.316148614 | 0.461004399 |
|  | DYL1 | 745.5931143 | 1452.311042 | 439.577402 | 238.4196081 |
|  | SAUR61 | 0 | 0.621693833 | 2.171080431 | 39.18582214 |
|  | SAR1 | 11.24893455 | 5.598960655 | 6.960902516 | 2.571412062 |
|  | AtMYB93 | 2.504098899 | 1.575246826 | 0.12312964 | 0 |
|  | AGO1 | 9.900970528 | 6.168914696 | 7.540501629 | 7.084682594 |
|  | IAA18 | 8.527313203 | 7.507000381 | 5.787012846 | 15.19277288 |
|  | AXR4 | 79.74410824 | 37.45056789 | 34.7900707 | 18.67656276 |
|  | ARF1 | 15.76639341 | 1.898205381 | 0.370934479 | 0.288476021 |
|  | RPN12A | 56.98613638 | 50.40789845 | 69.33957704 | 62.89249467 |
|  | ETR1 | 10.76303785 | 18.42770109 | 5.241915775 | 7.513041793 |
|  | MIF1 | 66.75478914 | 17.09800437 | 73.56458101 | 128.1896225 |
| CTK | ETN8 | 22.75827634 | 26.85478271 | 40.77754922 | 14.9492587 |
|  | DRB1 | 41.68636207 | 32.36236763 | 27.22247268 | 12.02285843 |
|  | ARR4 | 4.978967678 | 15.74780587 | 10.92779299 | 3.017633089 |
|  | IPT6 | 1.741052279 | 1.973072341 | 2.656640344 | 1.489111173 |
|  | AHK3 | 13.93817567 | 9.274925533 | 25.30705917 | 38.29191497 |
|  | BRX | 19.83914112 | 11.85494792 | 5.732371338 | 2.170456456 |
|  | ACC1 | 22.75770069 | 19.1698291 | 15.68603353 | 20.44674155 |
|  | GCR1 | 12.59132383 | 11.44114191 | 11.94989333 | 4.271358657 |
|  | STM | 38.21059962 | 54.37104656 | 6.449572953 | 11.71462094 |
|  | RPN12A | 56.98613638 | 50.40789845 | 69.33957704 | 62.89249467 |
|  | ARR11 | 4.154390584 | 3.894465648 | 3.925313571 | 2.18051378 |
|  | IPT1 | 6.026065081 | 3.030982601 | 4.947173599 | 2.486415203 |
|  | MIF1 | 66.75478914 | 17.09800437 | 73.56458101 | 128.1896225 |
|  | CKX5 | 2.879448353 | 2.111834401 | 4.483486209 | 4.87016807 |
|  | RR3 | 106.3346817 | 86.01320246 | 110.4232764 | 107.6361955 |
|  | RR1 | 13.19308127 | 24.19665712 | 15.73070153 | 10.88072006 |
|  | RR2 | 37.71185579 | 35.1795409 | 30.64467868 | 16.22168115 |
|  | RR5 | 2.853670498 | 20.79898975 | 32.61195655 | 10.91258079 |
|  | RR9 | 10.76311175 | 33.1994342 | 9.510899112 | 0.74562836 |
|  | RR11 | 26.52779772 | 59.9972135 | 59.63322358 | 72.37959151 |
|  | RR12 | 14.57840209 | 17.04858268 | 39.71619744 | 14.73770397 |
|  | QUA2 | 2.481547117 | 1.580332585 | 14.3261388 | 5.843109992 |
| GA | LHY | 10.19513443 | 8.559177832 | 12.22586063 | 11.61654491 |
|  | GA2OX6 | 14.29585221 | 16.46392236 | 15.72794594 | 14.55706737 |
|  | KAO1 | 5.234310048 | 10.78349403 | 13.88143683 | 17.37676981 |
|  | ATMYB13 | 11.03620842 | 11.99432304 | 27.91580836 | 43.98749264 |
|  | MYB60 | 21.04802767 | 14.23770603 | 46.34959393 | 39.37864116 |
|  | PIF3 | 8.526238349 | 10.78133715 | 6.996237914 | 2.536172032 |
|  | DREB1F | 14.28450957 | 1.076964002 | 0.210453034 | 1.329814536 |
|  | GAI | 15.41244358 | 7.932598538 | 17.27204127 | 6.684983172 |
|  | GA3OX1 | 0 | 0 | 0.939532685 | 1.405144749 |
|  | GASA9 | 6.614723586 | 12.71653802 | 0.921073668 | 0.223849899 |
|  | GI | 20.15446746 | 33.66424765 | 81.97428164 | 87.23685624 |
|  | GA2OX7 | 1.452614717 | 1.149781246 | 0.178197669 | 0.382379813 |
|  | LBD6 | 10.20433673 | 9.074302662 | 0.773186781 | 0.388010012 |
|  | ETR1 | 10.76303785 | 18.42770109 | 5.241915775 | 7.513041793 |
|  | RGL1 | 15.92137062 | 8.69716154 | 8.316900741 | 6.327449911 |
|  | MIF1 | 66.75478914 | 17.09800437 | 73.56458101 | 128.1896225 |
|  | GASA1 | 29.89558073 | 123.1289417 | 3.058965104 | 6.938631708 |
|  | GID1 | 4.37289417 | 1.13526932 | 0 | 0.099536721 |
|  | GASA1 | 10.99857266 | 12.50516776 | 4.999519609 | 2.967503163 |
|  | GASA2 | 357.1241479 | 409.110178 | 28.52947565 | 15.62139597 |
