# Supplementary material for: Transcriptome Analysis and Identification of Genes Associated with Floral Transition and Flower Development in Sugar Apple (Annona squamosa L.)
Source: Front Plant Sci. 2016 Nov 9;7:1695. doi: 10.3389/fpls.2016.01695 (PMC5101194; doi:10.3389/fpls.2016.01695)
Supplement: Supplementary file 7 [file Table7.DOCX]

Table S7 The primer sequences of 20 selected genes

| gene | UP | DN |
| --- | --- | --- |
| LEAFY | GGGAACTCCTCGTCGGCGA | CTTTCTACGCCGTTGCTG |
| AP2 | CTGCATGAATTGGATCAT | CTCAGTTCAATGAGTAGC |
| AP3 | CATTTCTCATTTCTACCATC | CCGAAACCTGCTGATAAC |
| CO | CAGAGTGGCTTCTCGCCA | TGCGCCTTTTCCTTCTCTG |
| COL | CACTCTACATCAATTGGC | GGAGCGGGACGTCGGGAAG |
| SEP1 | CCTAGCGGTTTCTCGTCT | TGGCTTCATTCCAGGGTGG |
| AGL6 | GGTCCAACTTGAACTTGG | GCTGGCTCTTGGGAAGTGC |
| AGL15 | AGAATCATCATTCTTCCC | CAAGCTTGAGTTTGGATGA |
| AGL62 | CAAGGTCTTCTCCTTCGGC | AACGGCATTGCCGGCGACC |
| SOC1 | GAGTAGGCAGGTCACGTT | CTTTCTTCCTCTAATGTTG |
| GI | CAGCAGCCACTACATAAG | GGCAATTCTACAACGAAC |
| EMF1 | GGACCGTAGCCGGCCAGCT | GAATTCTGTTACATGCAGCG |
| EMF2 | GAGATGAGTTATTTTACAAG | GCATAGCTAATTTTATAAG |
| GA2ox | CAAAAAACAGCAGCATC | CACCAGTGATGGGAGTTG |
| GA3ox | CAAGTCATCAACCACGGA | TAGCTAGCTAGGCAGAAGAT |
| GA20ox | GTTAATAATTGTTGTACC | TTTCCTCAACTTCACCCAA |
| FCA | CAGAGGCGGATCTCCTGA | CAGCATACCTAACTTGAATGG |
| FPA | GTAAAATGAGCATCTCTG | CTGGCTAGAATTTGAATTTC |
| ARP6 | CTACACGGATGCATACA | CTGTTAATTCTTGTCATC |
| SPL9 | CAGATAGGTCCAAAGTCCC | TTCTCAGCAATGGGACCCC |
