# Supplementary material for: Transcriptome Analysis and Identification of Genes Associated with Floral Transition and Flower Development in Sugar Apple (Annona squamosa L.)
Source: Front Plant Sci. 2016 Nov 9;7:1695. doi: 10.3389/fpls.2016.01695 (PMC5101194; doi:10.3389/fpls.2016.01695)
Supplement: Supplementary file 8 [file Table8.DOCX]

Table S8 Validation of the expression of flowering-related Genes in sugar apple.

| Gene name | Unigene ID | IM | FB | FL1 | FL2 |
| --- | --- | --- | --- | --- | --- |
| LEAFY | Unigene0071901 | 8.432577256 | 1.138247953 | 0.34275548 | 0.135809131 |
| AP2 | Unigene0032833 | 49.43289687 | 40.03477322 | 18.5562983 | 20.13587268 |
| AP3 | Unigene0026260 | 183.8787759 | 173.0682269 | 30.4760107 | 8.965160334 |
| CO | Unigene0055214 | 3.705425271 | 1.831735034 | 1.29237227 | 0.052940689 |
| COL | Unigene0020378 | 31.00660801 | 14.56390004 | 4.17915706 | 2.473033118 |
| SEP1 | Unigene0029534 | 70.56640744 | 60.45375227 | 79.2930552 | 63.29614432 |
| AGL6 | Unigene0041309 | 82.07128832 | 62.74134655 | 35.1673744 | 19.85352435 |
| AGL15 | Unigene0038024 | 1.763013439 | 4.300844281 | 10.2748123 | 14.44564732 |
| AGL62 | Unigene0065297 | 5.816537699 | 1.116460002 | 1.50001348 | 0.592529771 |
| SOC1 | Unigene0018025 | 5.342333172 | 7.378028634 | 0.44277265 | 0.702264127 |
| GI | Unigene0035917 | 36.47797609 | 70.04461698 | 173.393154 | 193.7420418 |
| EMF1 | Unigene0038483 | 5.822009198 | 7.830555253 | 5.19257126 | 2.767417336 |
| EMF2 | Unigene0051363 | 1.141722791 | 0.897336032 | 0 | 0 |
| GA2ox | Unigene0063968 | 2.618554453 | 36.88116839 | 1.42147837 | 2.862413426 |
| GA3ox | Unigene0027158 | 0.855097317 | 1.357835497 | 5.80225081 | 72.77255728 |
| GA20ox | Unigene0019607 | 10.62151183 | 15.08434977 | 1.95037948 | 0.977758671 |
| FCA | Unigene0039749 | 38.96016886 | 23.93578334 | 24.8934527 | 14.11374299 |
| FPA | Unigene0016124 | 18.74223926 | 15.56779819 | 11.4967234 | 5.299281314 |
| ARP6 | Unigene0012497 | 10.57100983 | 1.908330328 | 2.15215439 | 0.734193073 |
| SPL9 | Unigene0014208 | 24.18747003 | 15.26143621 | 2.55402384 | 0.997660318 |
