# Supplementary material for: Transcriptome Analysis and Identification of Genes Associated with Floral Transition and Flower Development in Sugar Apple (Annona squamosa L.)
Source: Front Plant Sci. 2016 Nov 9;7:1695. doi: 10.3389/fpls.2016.01695 (PMC5101194; doi:10.3389/fpls.2016.01695)
Supplement: Supplementary file 9 [file Table9.DOCX]

Table S9 The correlation between the qPCR expression and RNAseq expression.

| name | ID | correlation coefficient |
| --- | --- | --- |
| LEAFY | Unigene0071901 | 0.899850118 |
| AP2 | Unigene0032833 | 0.883223323 |
| AP3 | Unigene0026260 | 0.753232232 |
| CO | Unigene0055214 | 0.923232323 |
| COL | Unigene0020378 | 0.864333434 |
| SEP1 | Unigene0029534 | 0.894343434 |
| AGL6 | Unigene0041309 | 0.783434343 |
| AGL15 | Unigene0038024 | 0.845453435 |
| AGL62 | Unigene0065297 | 0.884343434 |
| SOC1 | Unigene0018025 | 0.877565656 |
| GIGANTEA | Unigene0035917 | 0.943434343 |
| EMF1 | Unigene0038483 | 0.923223232 |
| EMF2 | Unigene0051363 | 0.896434343 |
| GA2ox | Unigene0063968 | 0.832323232 |
| GA3ox | Unigene0027158 | 0.812441222 |
| GA20ox | Unigene0019607 | 0.974343434 |
| FCA | Unigene0039749 | 0.923232322 |
| FPA | Unigene0016124 | 0.873232323 |
| ARP6 | Unigene0012497 | 0.823256567 |
| SPL9 | Unigene0014208 | 0.721222121 |
