# Supplementary figures and images for: Transcriptome Analysis and Identification of Genes Associated with Floral Transition and Flower Development in Sugar Apple (Annona squamosa L.)
Source: Front Plant Sci. 2016 Nov 9;7:1695. doi: 10.3389/fpls.2016.01695 (PMC5101194; doi:10.3389/fpls.2016.01695)

Fig, S1 Length distribution of sugar apple (*Annina squamosa*) unigenes.

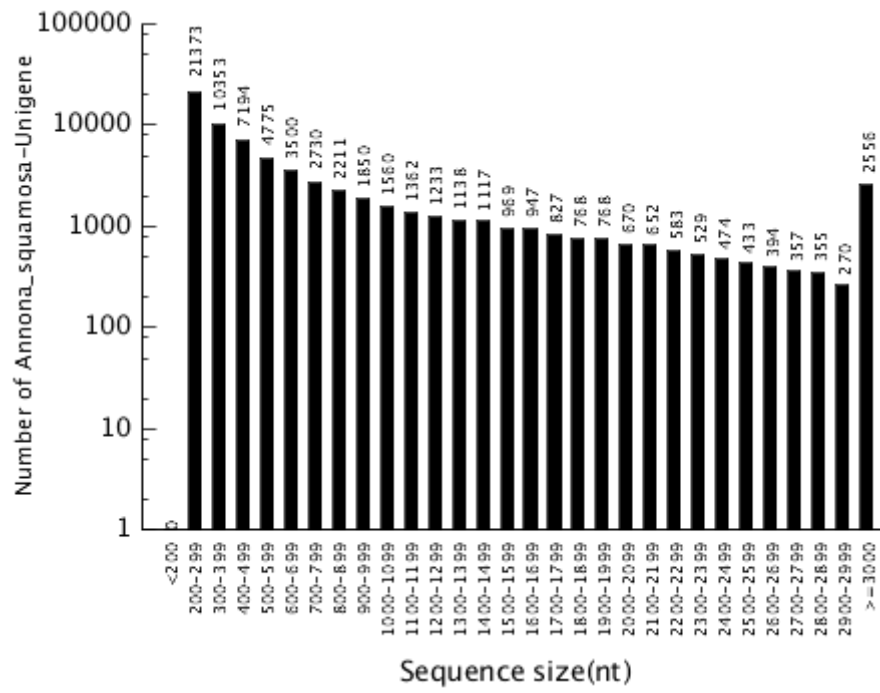

Supplement: Supplementary file 10 [file Image1.PDF]

Fig. S2 GO class of sugar apple unigenes

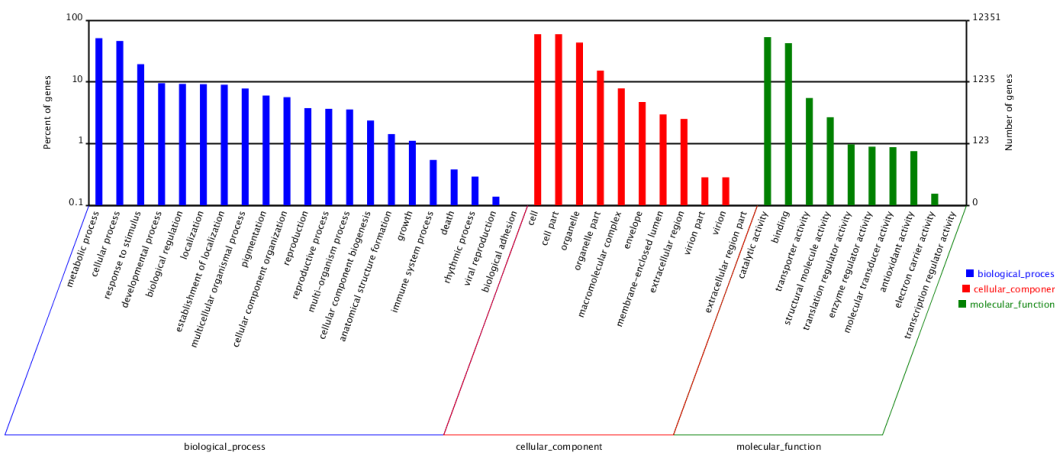

Supplement: Supplementary file 11 [file Image2.PDF]

Fig. S3 Identification of flower developmental stage-specific expressed genes.

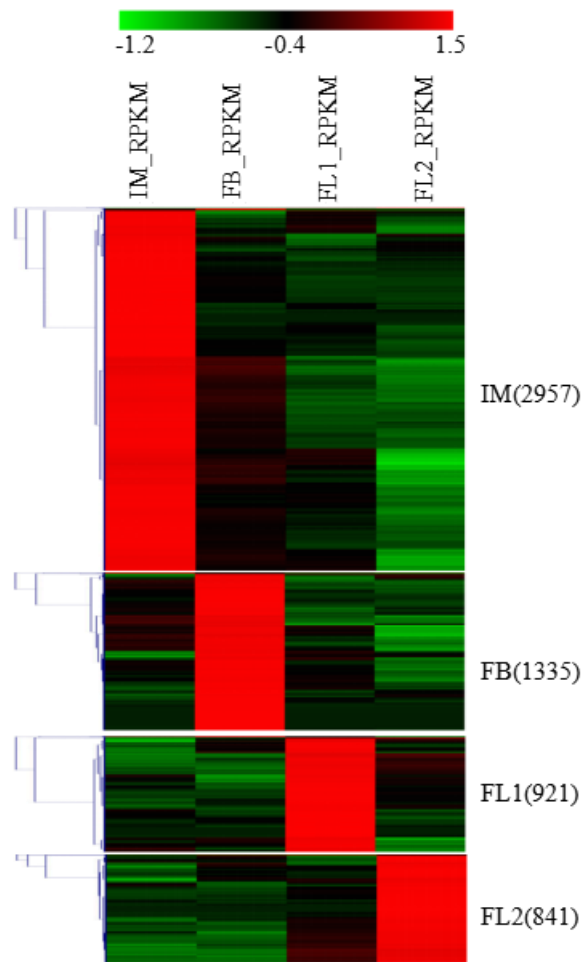

Supplement: Supplementary file 12 [file Image3.PDF]
